# Supplementary figures and images for: GRK2 kinases in the primary cilium initiate SMOOTHENED-PKA signaling in the Hedgehog cascade
Source: PLoS Biol. 2024 Aug 13;22(8):e3002685. doi: 10.1371/journal.pbio.3002685 (PMC11322411; doi:10.1371/journal.pbio.3002685)

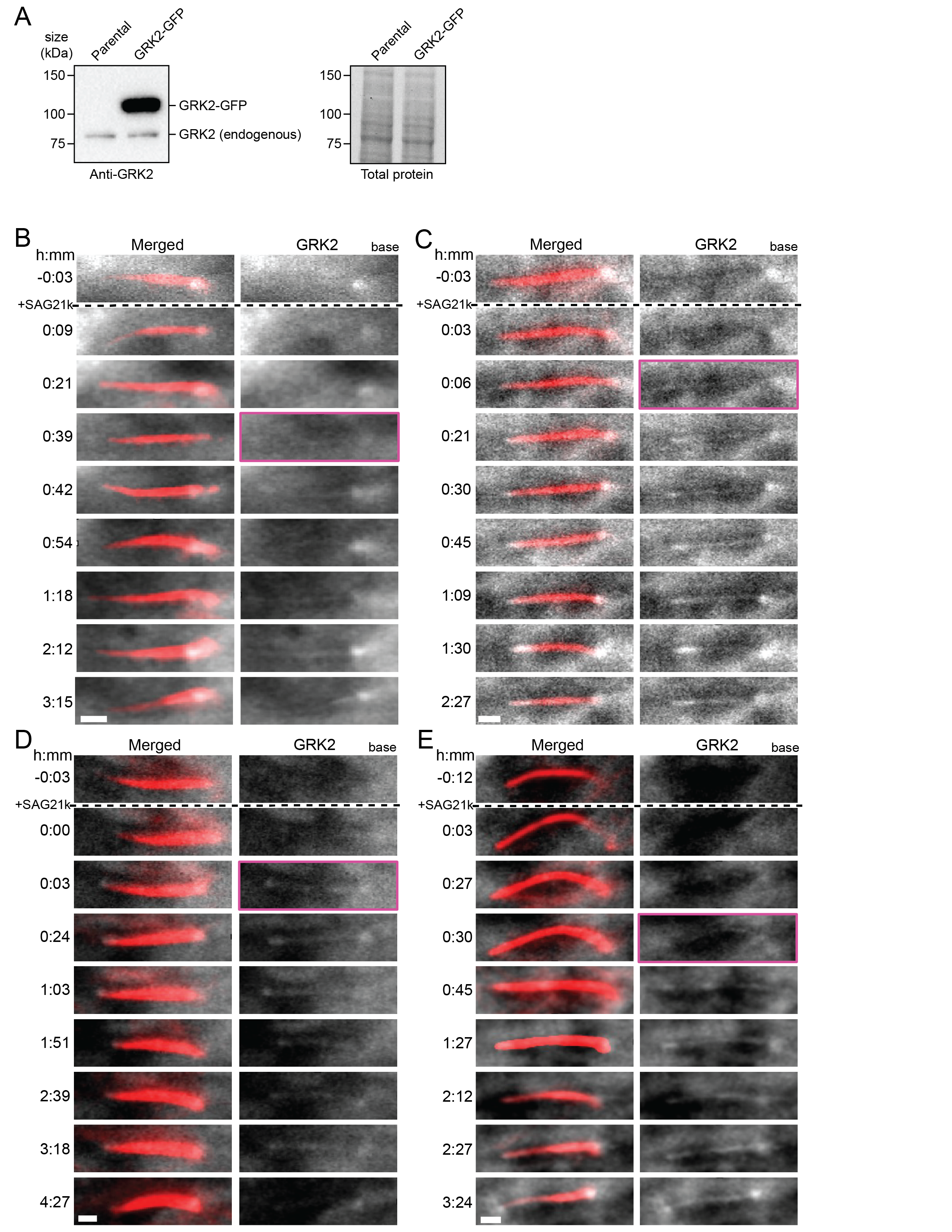

Supplement: S1 Fig — (A) Expression of the GRK2-eGFP fusion was verified via immunoblot analysis in IMCD3 Flp-in cells stably expressing the GRK2-eGFP fusion, revealing that the GRK2-eGFP was expressed at 23.99 +/- 0.06-fold higher levels relative to endogenous GRK2. Note that the GRK2-eGFP fusion runs at a higher molecular mass, due to the eGFP tag. Left: anti-GRK2 immunoblot; right: total protein (Stain Free imaging). (B–E) Four additional examples of cells showing SMO activation-induced localization of GRK2-eGFP to the ciliary shaft, monitored by TIRF imaging. Merged (SiR-Tubulin (red) to mark cilium and GRK2-eGFP (white) images) and GRK2-eGFP channels are shown, as in Fig 1C. Scale bar is as in Fig 1C. The uncropped protein gels and westerns are included in S1 Raw Images. (PNG) [file pbio.3002685.s002.png]

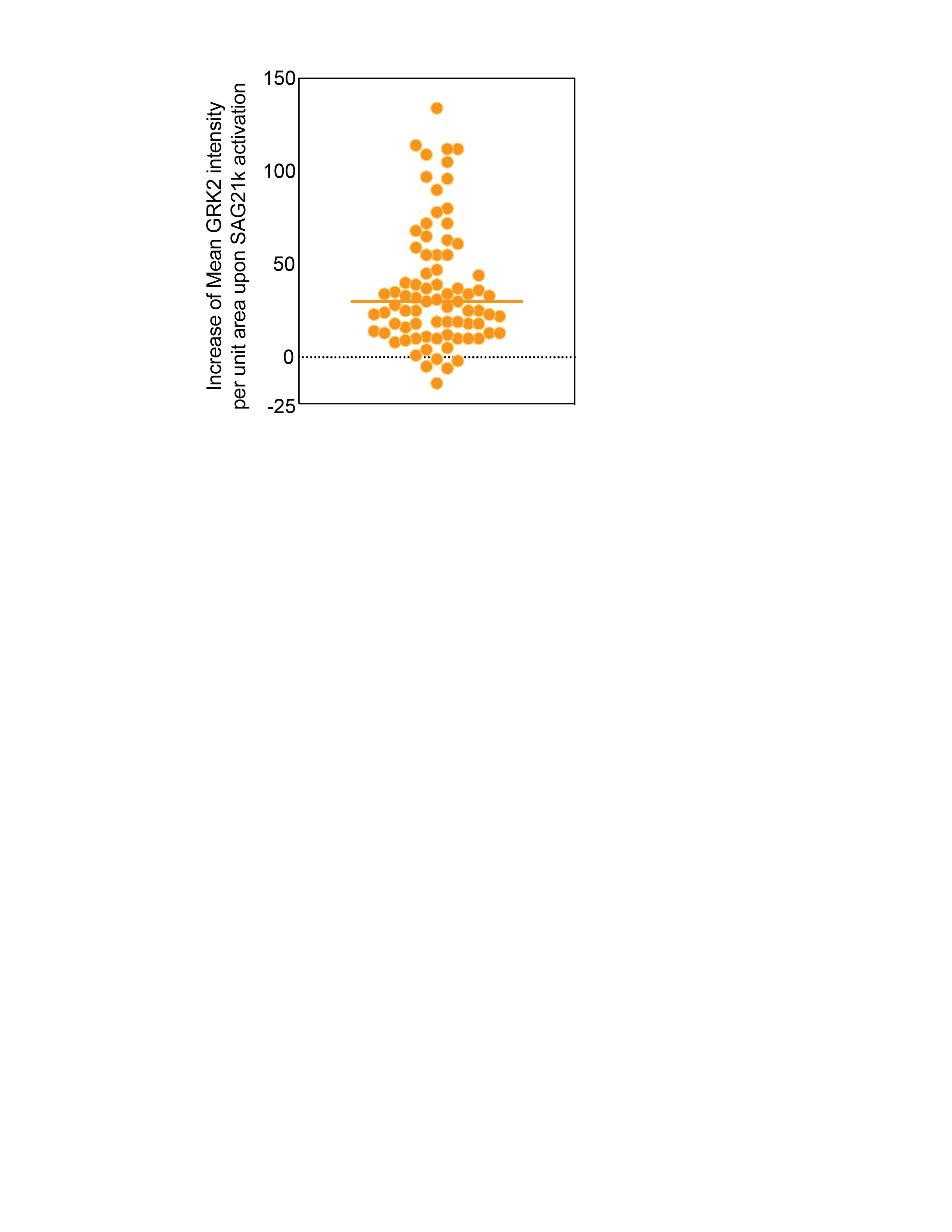

Supplement: S2 Fig — Scatterplot indicating the increase in mean GRK2 intensity per unit area within the cilium shaft following SAG21k activation. The dashed line in the plot represents the median value. The underlying data for this figure can be found under S1 Data. (PNG) [file pbio.3002685.s003.png]

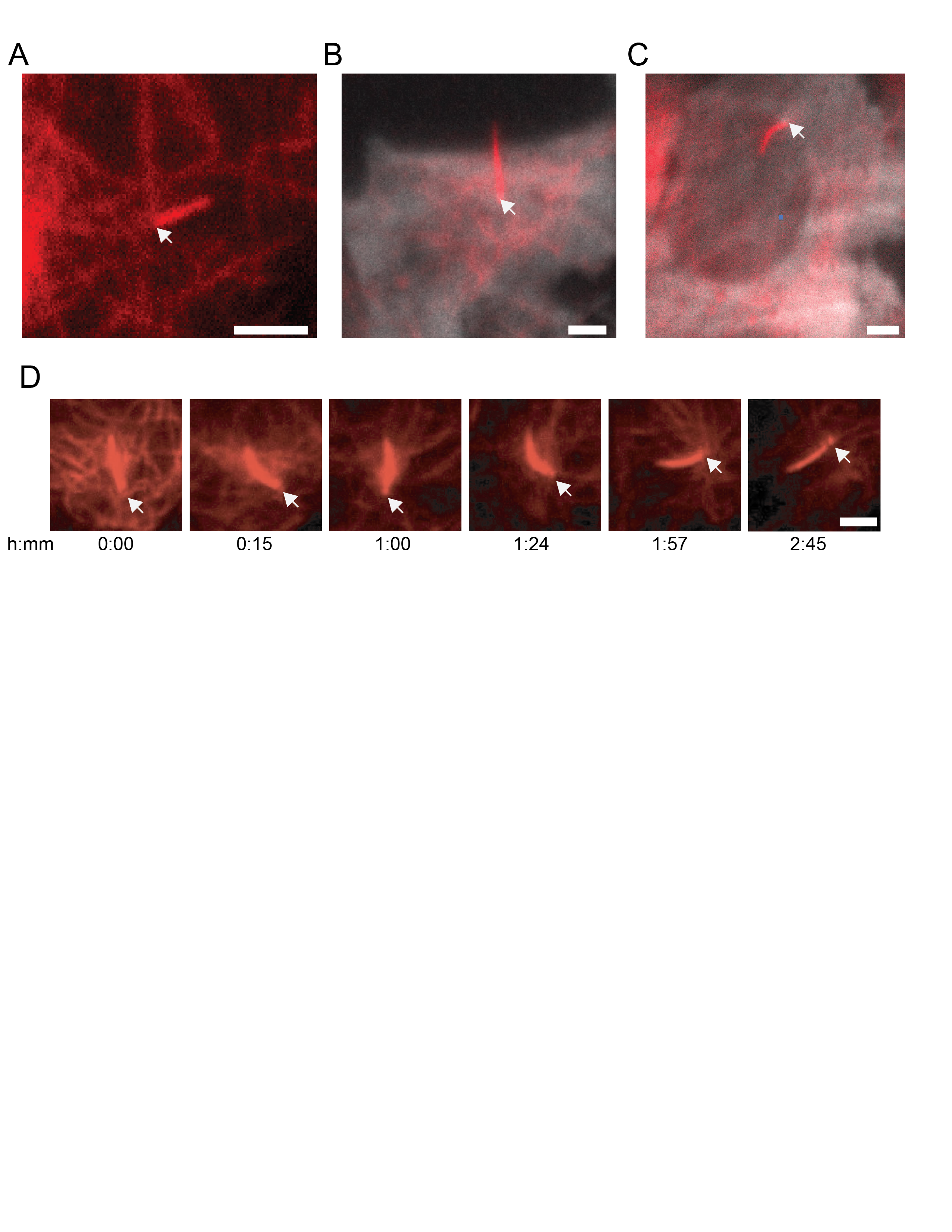

Supplement: S3 Fig — To distinguish the cilium base from the tip, several methodologies are employed. (A) The application of SiR-Tubulin dye, which stains both cytoplasmic microtubules and the ciliary axoneme, facilitates the visual identification of tubulin fibers converging from the cellular cytoplasm to the base of the cilia. In many instances, (B) cilia either extend from the cellular body into the extracellular space or (C) emanate from the nuclear periphery. (D) In cases where the determination of cilia base positioning is not apparent from an initial image, we followed the cilium over multiple time points; in many cases, such montages depict tubulin fibers converging towards the cilium base at later time points, enabling us to visualize the centriole (last image) and thereby distinguish the base from the tip. (Arrow points to the cilium base. Scale bar = 3 μm). (PNG) [file pbio.3002685.s004.png]

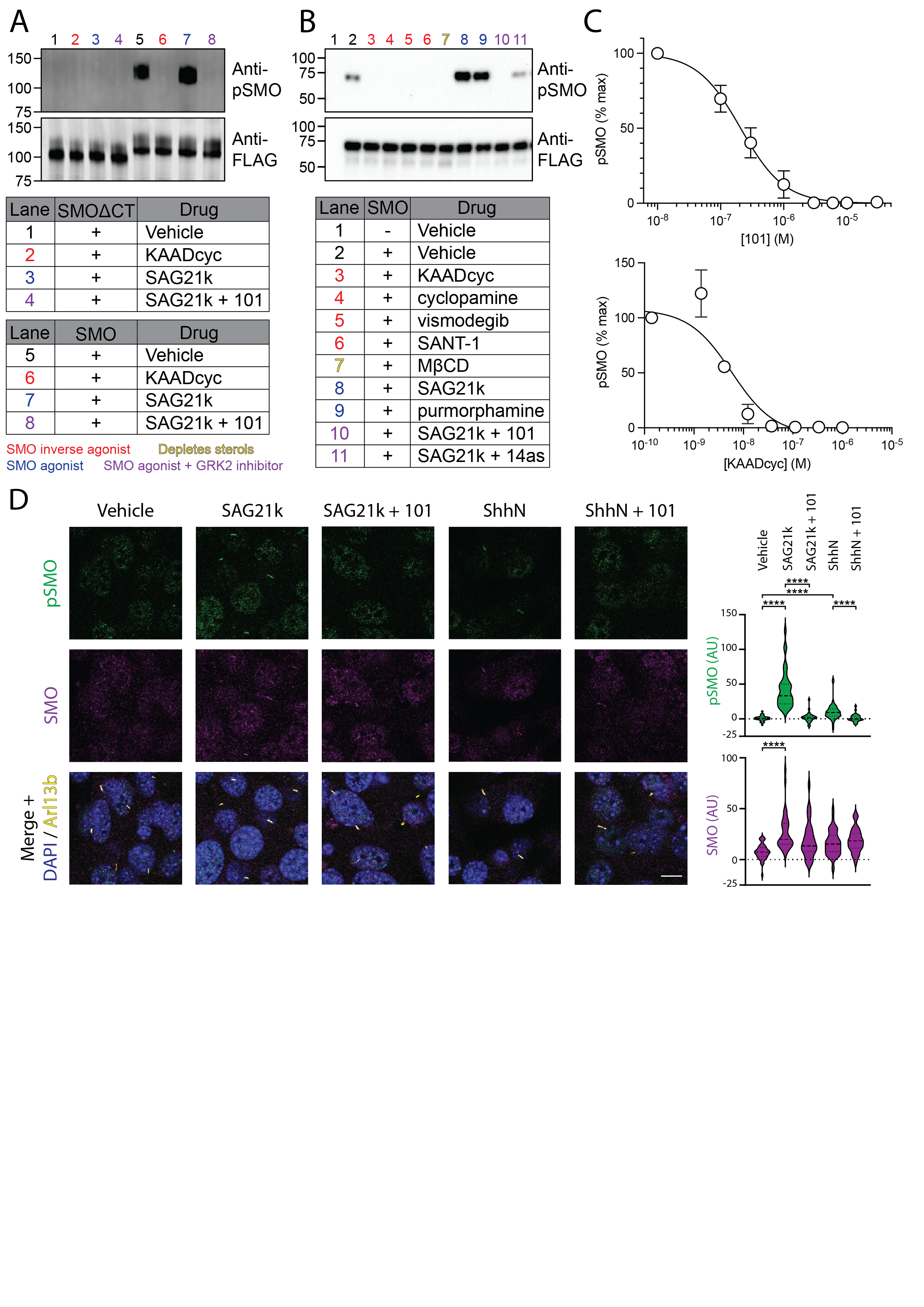

Supplement: S4 Fig — (A) HEK293 cells expressing GRK2-GFP along with C-terminal Gαo fusions of FLAG-tagged wild-type (WT) SMO or an SMO truncation mutant lacking all GRK2 phosphorylation sites (SMOΔCT) (see “Methods”) were treated for 4 h with vehicle, the SMO agonist SAG21k (1 μm), the SMO inverse agonist KAADcyc (1 μm), or SAG21k plus the GRK2 inhibitor Compound101 (101, 30 μm). SMO was then purified using FLAG affinity chromatography, and FLAG eluates were analyzed via western blot with anti-pSMO or anti-FLAG antibodies to probe phosphorylated SMO and total SMO, respectively. (B) HEK293 cells expressing GRK2-GFP and wild-type SMO (or a vector control) were treated with the indicated SMO inverse agonists (red), agonists (blue), agonist + GRK2 inhibitor (purple), or depleted of sterols using the cholesterol extracting agent methyl-β-cyclodextrin (MβCD, yellow), then analyzed as described in (A). Note that SMO overexpressed in HEK293 cells has substantial basal activity, as it exceeds regulation by endogenous PTCH1 [105–107,134] and is therefore constitutively bound to membrane sterols [27,107]. Consistent with this hypothesis, the anti-pSMO signal in vehicle-treated cells is absent in MβCD-treated cells. (C) Concentration-response analysis for blockade of SMO phosphorylation by KAADcyc or 101 revealed IC50 values of 5.8 nM and 207.7 nM, respectively, close to previously published values from Hh pathway transcriptional reporter assays [44,55]. (D) NIH3T3 cells were treated overnight with SAG21k (500 nM) or the N-terminal signaling domain of Sonic hedgehog (ShhN) (supplied as conditioned medium), in the presence or absence of 101 (30 μm), or with a vehicle control. Cells then were stained with anti-pSMO (green) to view phosphorylated SMO, anti-SMO (magenta) to view total SMO levels, and anti-Arl13b (yellow) to view primary cilia. Quantification of pSMO and SMO signals is shown at right. Significance in (D) was determined via a Mann–Whitney test. ****, p < 0.0001. n = 34–64 individual cil [file pbio.3002685.s005.png]

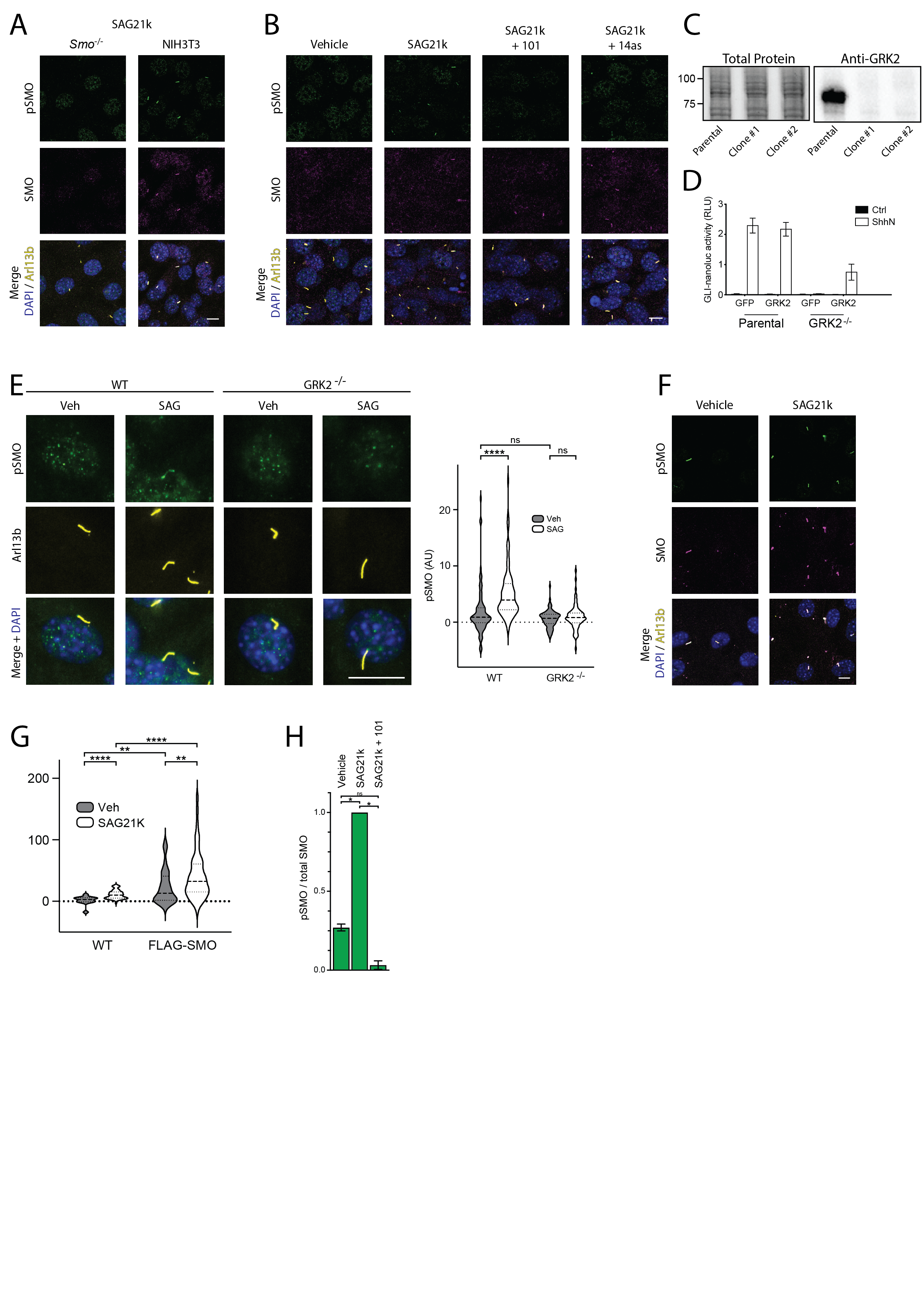

Supplement: S5 Fig — (A) Smo-/- mouse embryonic fibroblasts (MEFs) or NIH3T3 cells were treated with SAG21k, then stained as described in S4D Fig. Note that the anti-pSMO antibody recognizes both a ciliary and a nuclear antigen. Only the latter is present in Smo-/- MEFs, indicating that the nuclear signal is nonspecific and arises from a non-SMO antigen. (B) Effects of 2 independent GRK2 inhibitors, 101 and 14as, on SAG21k-induced SMO phosphorylation in NIH3T3 cells treated and stained as in (A). (C) Absence of GRK2 protein in NIH3T3 Grk2-/- cells was verified by immunoblotting. Lysates from 2 independent Grk2-/- clones are shown. (D) NIH3T3 parental or Grk2-/- cells were transfected with a GRK2 expression plasmid or a GFP control, and Hh pathway activity in response to ShhN (white) vs. control (black) conditioned medium was monitored via a GLI transcriptional reporter assay (see Methods). Note that Grk2-/- cells fail to respond to ShhN, and pathway responsiveness is restored when GRK2 (but not a GFP control) is reintroduced via transfection, consistent with previous findings [43,44]. (E) Wild-type or Grk2-/- NIH3T3 cells were treated for 4 h with SAG vs. a vehicle control, then stained with anti-pSMO, Arl13b, and DAPI as in (A). (F) NIH3T3 cells stably expressing FLAG-SMO were treated with SAG21k or a vehicle control, then stained with anti-pSMO (green), anti-FLAG (to detect total SMO, magenta), and anti-Arl13b (yellow). Scale bar = 10 μm in all images. (G) Expression levels of the stably overexpressed SMO were quantifed relative to endogenous SMO via ciliary immunofluorescence staining in NIH3T3 Flp-in parental vs. FLAG-SMO-expressing cell lines with anti-SMO antibody, revealing that SMO is overexpressed 2.83-fold relative to the endogenous protein. (H) Quantification of the immunoblot data in Fig 2D (mean +/- standard deviation from 2 independent experiments). Significance was determined via one-way ANOVA. *, p < 0.05; ns, not significant. The underlying data for this figure can be f [file pbio.3002685.s006.png]

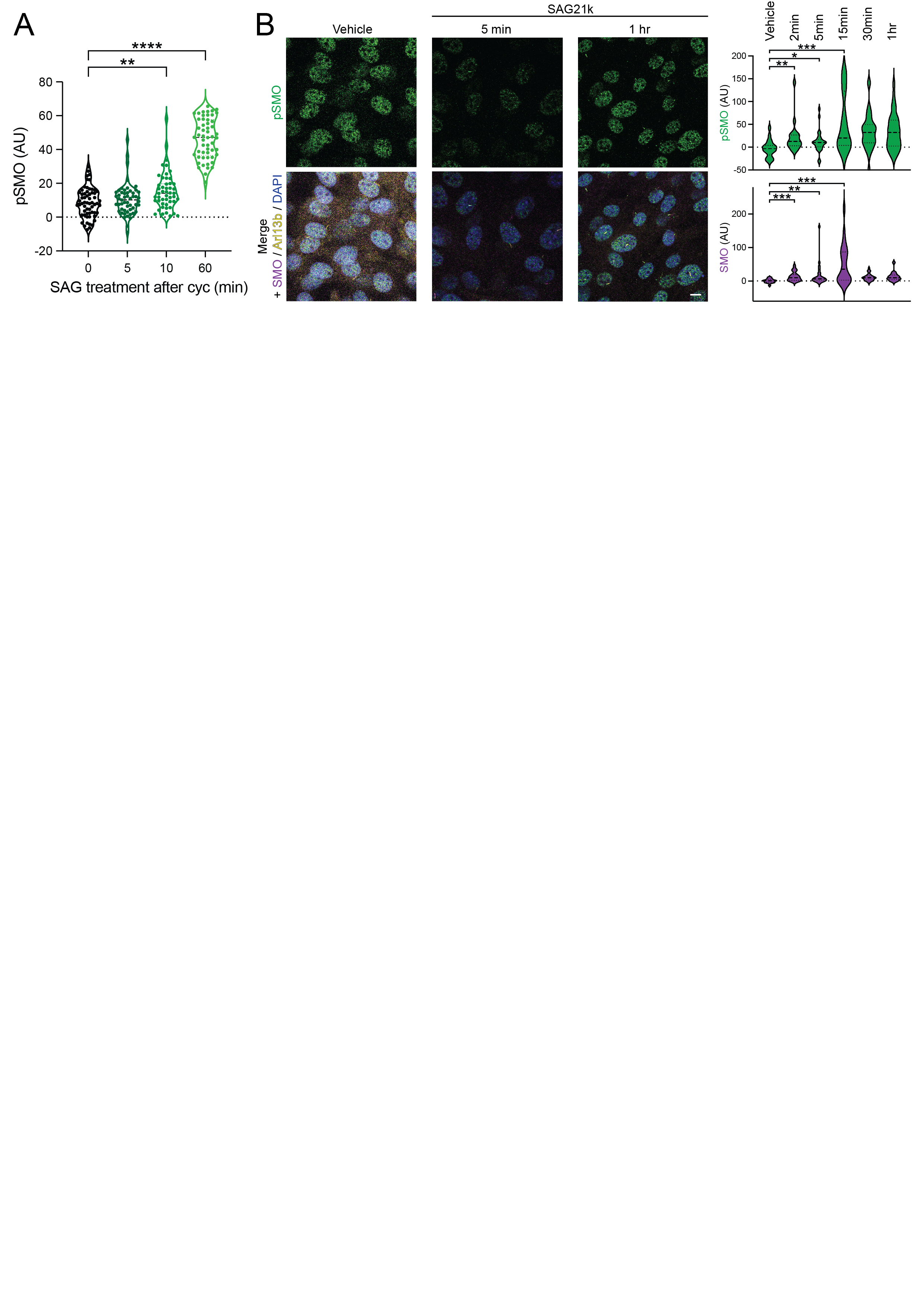

Supplement: S6 Fig — (A) Quantification of pSMO intensities in individual cilia from the NIH3T3 cells pretreated with cyclopamine and treated with SAG in Fig 3A. (B) NIH3T3 dync2 cells were treated with SAG21k for the indicated times, then processed and stained as described in Fig 2C. Quantification is shown at right. Note that the anti-pSMO antibody specifically recognizes phosphorylated SMO in cilia but also nonspecifically stains a nuclear antigen (see S5A Fig). Significance in (A) was determined as in Fig 3A, and in (B) was determined via a Mann–Whitney test. *, p < 0.05; **, p < 0.01; ***, p < 0.001; ****, p < 0.0001. n = 20–43 individual cilia per condition. Scale bar = 10 μm. The underlying data for this figure can be found under S3 Data. (PNG) [file pbio.3002685.s007.png]

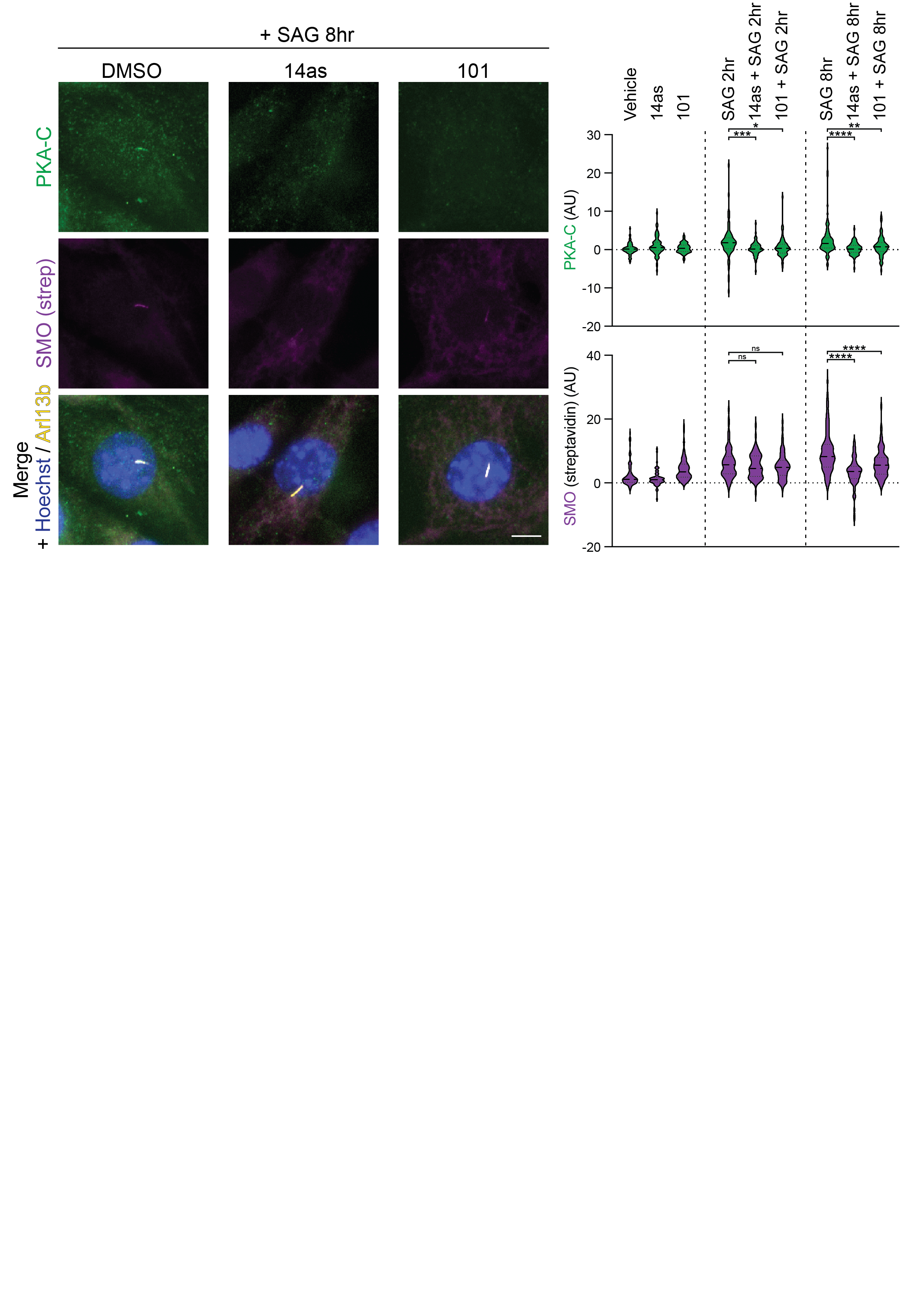

Supplement: S7 Fig — The complete experiment from Fig 5B, in which NIH3T3 cells stably expressing SMO-V5-TurboID were treated with vehicle, Cmpd101 or 14as for 16 h. SAG was then added to the culture medium in the presence of the indicated inhibitors and incubated for 2 h or 8 h. Cells were labeled with biotin for 10 min before fixation. For simplicity, the 2-h time point is presented in the main figure panel, and the complete experiment including all time points is presented here. Note that while the GRK2 inhibitors abolish ciliary SMO/PKA-C colocalization at 2 h without affecting total SMO levels in cilia, these inhibitors did block the further accumulation of SMO in cilia between 2 h and 8 h, suggesting that prolonged GRK2 inhibition may affect SMO ciliary trafficking under some conditions. Such effects, however, cannot account for the effects of GRK2 inhibitors on SMO/PKA-C localization at the earlier time point, leading to the conclusion that these inhibitors block SMO/PKA-C interaction in cilia primarily by directly disrupting the SMO/PKA-C complex, rather than by affecting SMO ciliary accumulation. Significance was determined as in Fig 5. *, p <0.05; **, p < 0.01; ***, p < 0.001; ****, p < 0.0001. n = 90–100 individual cilia per condition. Scale bar = 5 μm in all images. The underlying data for this figure can be found under S5 Data. (PNG) [file pbio.3002685.s008.png]

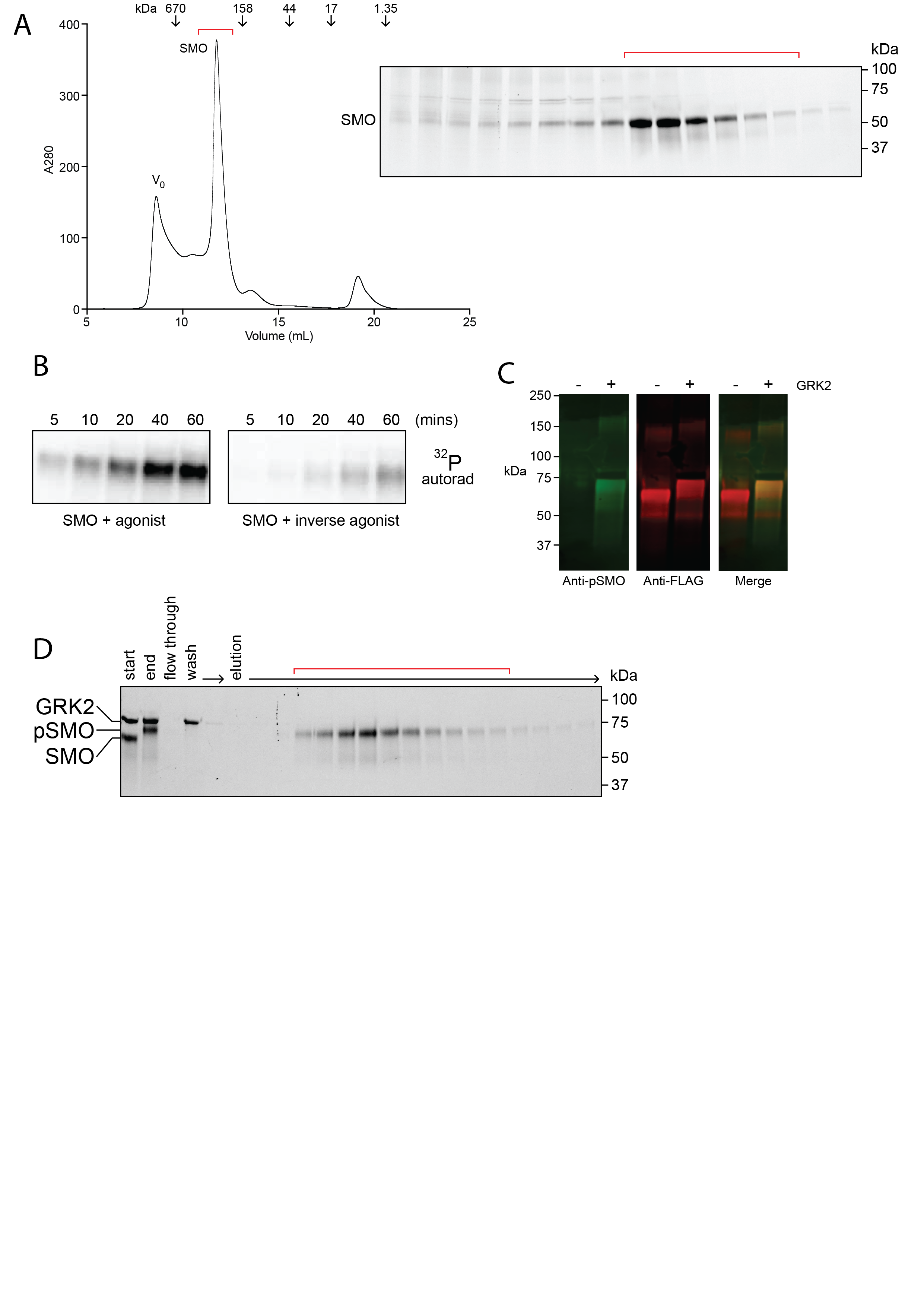

Supplement: S8 Fig — (A) Size exclusion chromatography of FLAG-SMO affinity-purified from HEK293 cells. Positions of the void (V0) and molecular weight standards are indicted. The peak corresponding to monodisperse SMO (red bracket) was collected and used for in vitro reconstitution studies. SDS-PAGE analysis of monodisperse SMO fractions is shown at right. (B) Phosphorylated agonist- or inverse-agonist loaded SMO was prepared as in Fig 6B but in the presence of γ32P-ATP, then analyzed by autoradiography. (C) Immunoblot analysis of purified SMO before and after the GRK2 phosphorylation reaction, demonstrating phosphorylation at the GRK2 cluster recognized by our anti-pSMO antibody. (D) Following GRK2 phosphorylation, pSMO was separated from GRK2 via FLAG affinity chromatography. Flow-through, wash, and elution fractions from the purification procedure are analyzed by SDS-PAGE. “Start” and “end” correspond to GRK2 phosphorylation reaction prior to addition of ATP (“start”) or after completion of the reaction (“end”). Red brackets indicate elution fractions that were collected, pooled, and used for subsequent experiments. Positions of SMO, pSMO, and GRK2 are indicated at left. The underlying data for this figure can be found under S6 Data. The uncropped protein gels and westerns are included in S1 Raw Images. (PNG) [file pbio.3002685.s009.png]

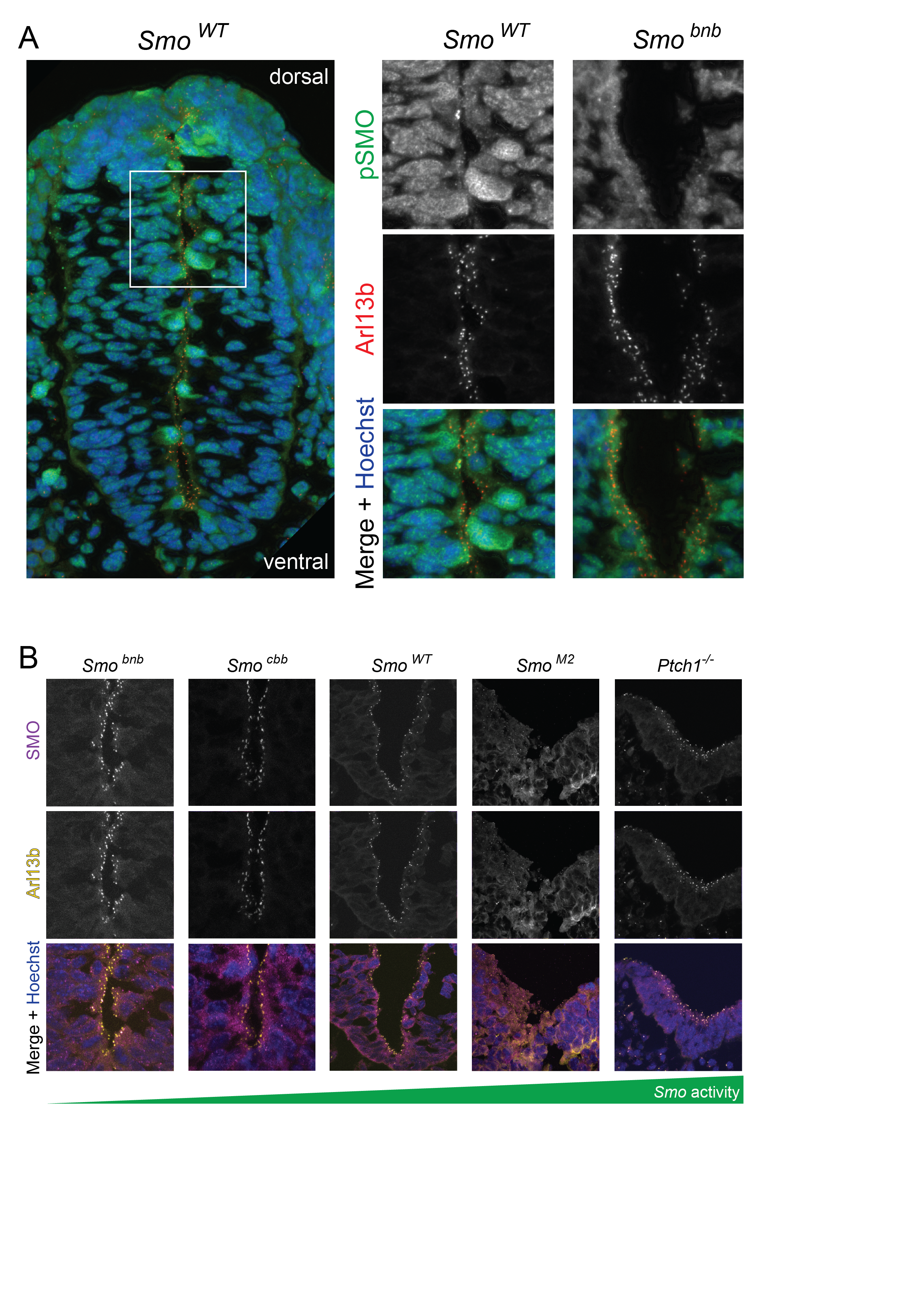

Supplement: S9 Fig — (A) Neural tubes from the indicated wild-type or Smobnb mutant E9.5 mice (low-magnification view of wild-type mouse at left, higher-magnification view at right, boxed region indicates the zoomed-in portion) were stained for pSMO and Arl13b, as in Fig 7A. Images are oriented with dorsal pointing up and ventral pointing down. (B) Neural tubes from the indicated genotypes were stained for total SMO (magenta), Arl13b (yellow), and nuclei (Hoechst, blue). (PNG) [file pbio.3002685.s010.png]

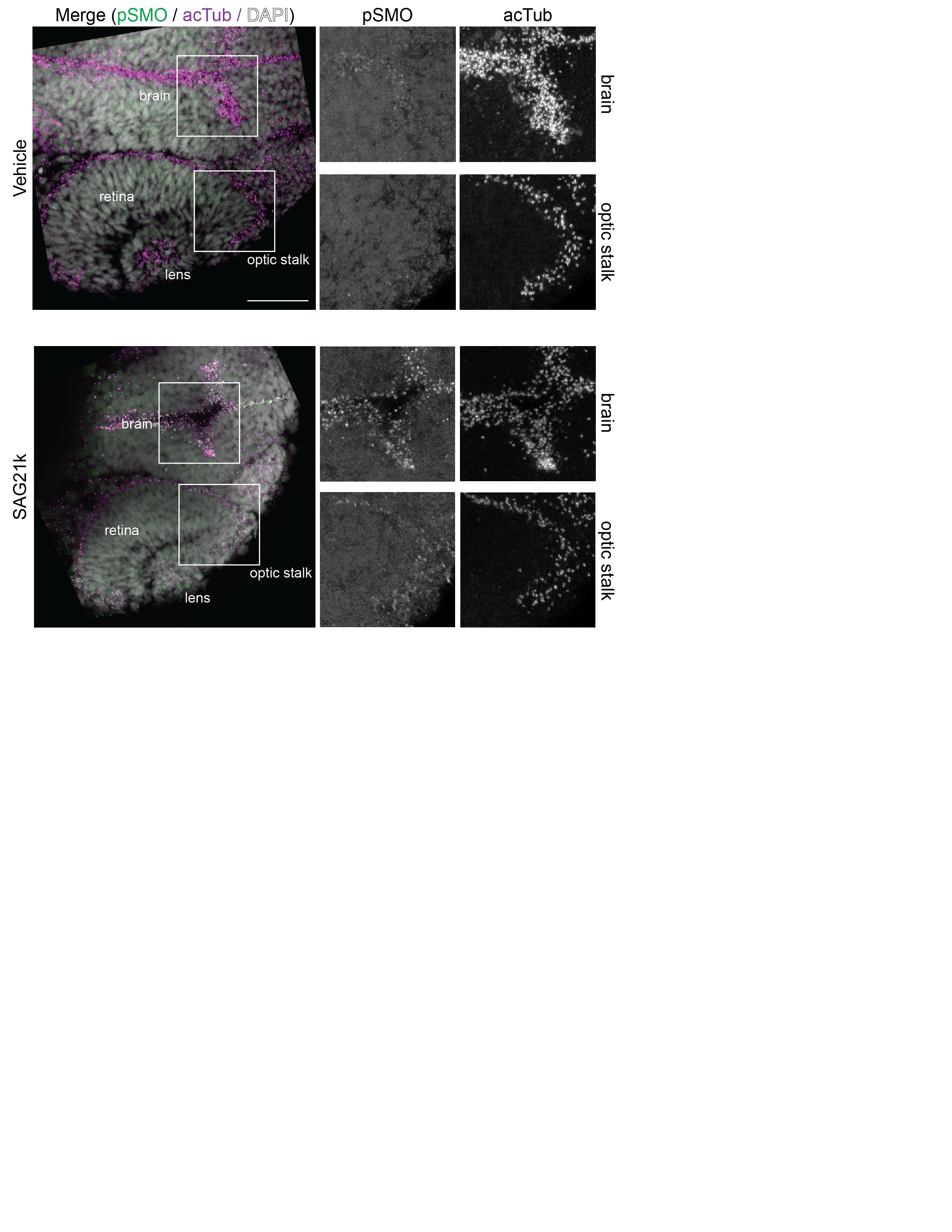

Supplement: S10 Fig — Vehicle (DMSO) or SAG21k-treated (10 μm) zebrafish embryos were stained for pSmo (green), acetylated tubulin (acTub, magenta), and nuclei (DAPI, white). Locations of brain and lens are indicated with boxes. Merged image is shown at left, and zoomed-in views of individual channels within the boxed regions are shown at right. pSmo sparsely labels acetylated tubulin-positive punctae in vehicle-treated embryos, while SAG21k-treated embryos show extensive cilia labeling. All embryos are 24 h postfertilization, dorsal view. Scale bar, 50 μm. (PNG) [file pbio.3002685.s011.png]

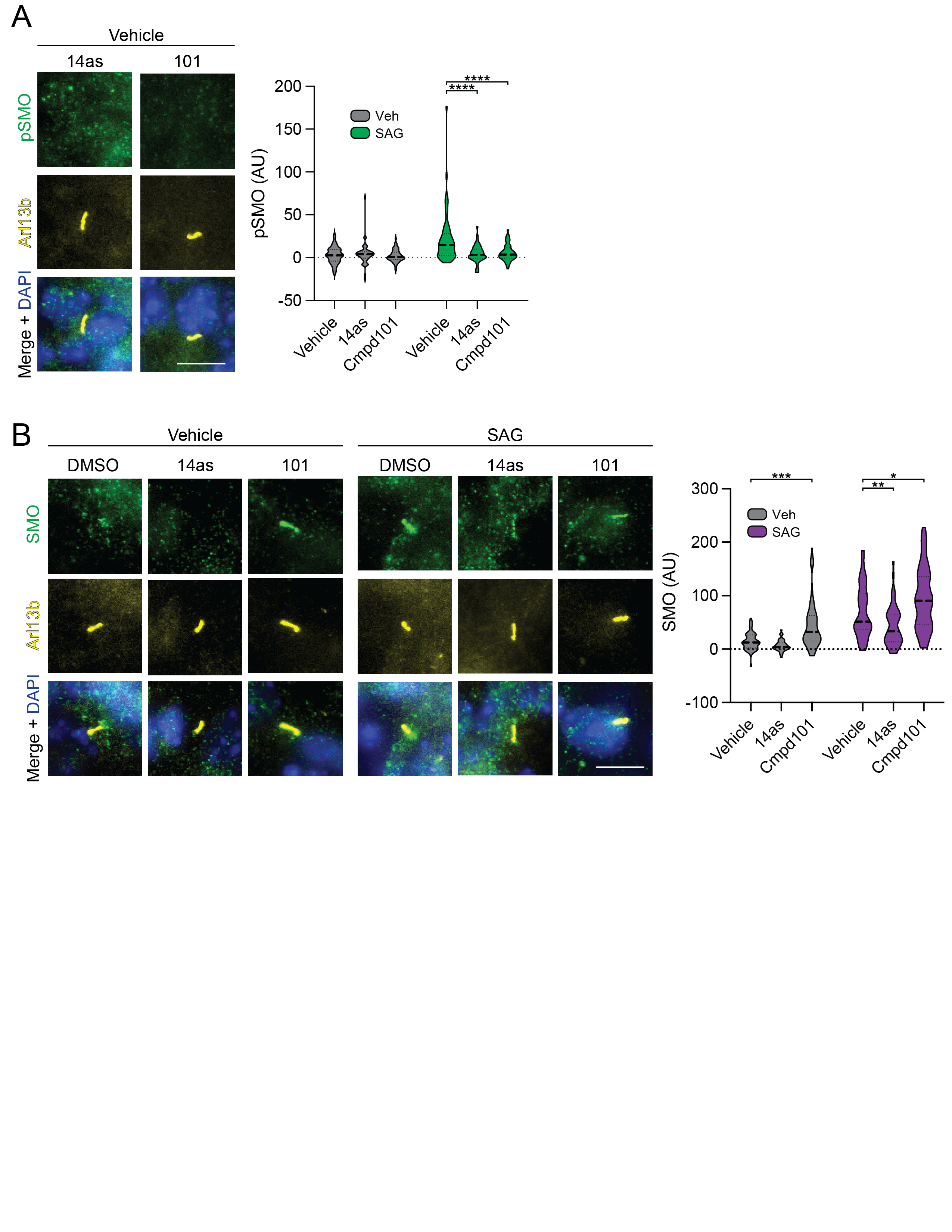

Supplement: S11 Fig — (A) Additional raw images from the experiment in Fig 7C, in which cells were treated with 14as or Cmpd101 in the absence of SAG, then stained for pSMO as described in the main figure. Quantification of the complete experiment is shown at right (B) CGNPs treated as in Fig 7C were stained for total SMO (green), Arl13b (yellow), and DAPI (blue). Quantification is shown at right. *, p < 0.05; **, p < 0.01; ***, p < 0.001; ****, p < 0.0001. Scale bar = 10 μm. The underlying data for this figure can be found under S7 Data. (PNG) [file pbio.3002685.s012.png]
