# Supplementary material for: GRK2 kinases in the primary cilium initiate SMOOTHENED-PKA signaling in the Hedgehog cascade
Source: PLoS Biol. 2024 Aug 13;22(8):e3002685. doi: 10.1371/journal.pbio.3002685 (PMC11322411; doi:10.1371/journal.pbio.3002685)
Supplement: S1 Raw Images — (PDF) [file pbio.3002685.s020.pdf]

Fig S1
Anti-GRK2

1 parental
2 GRK2-eGFP

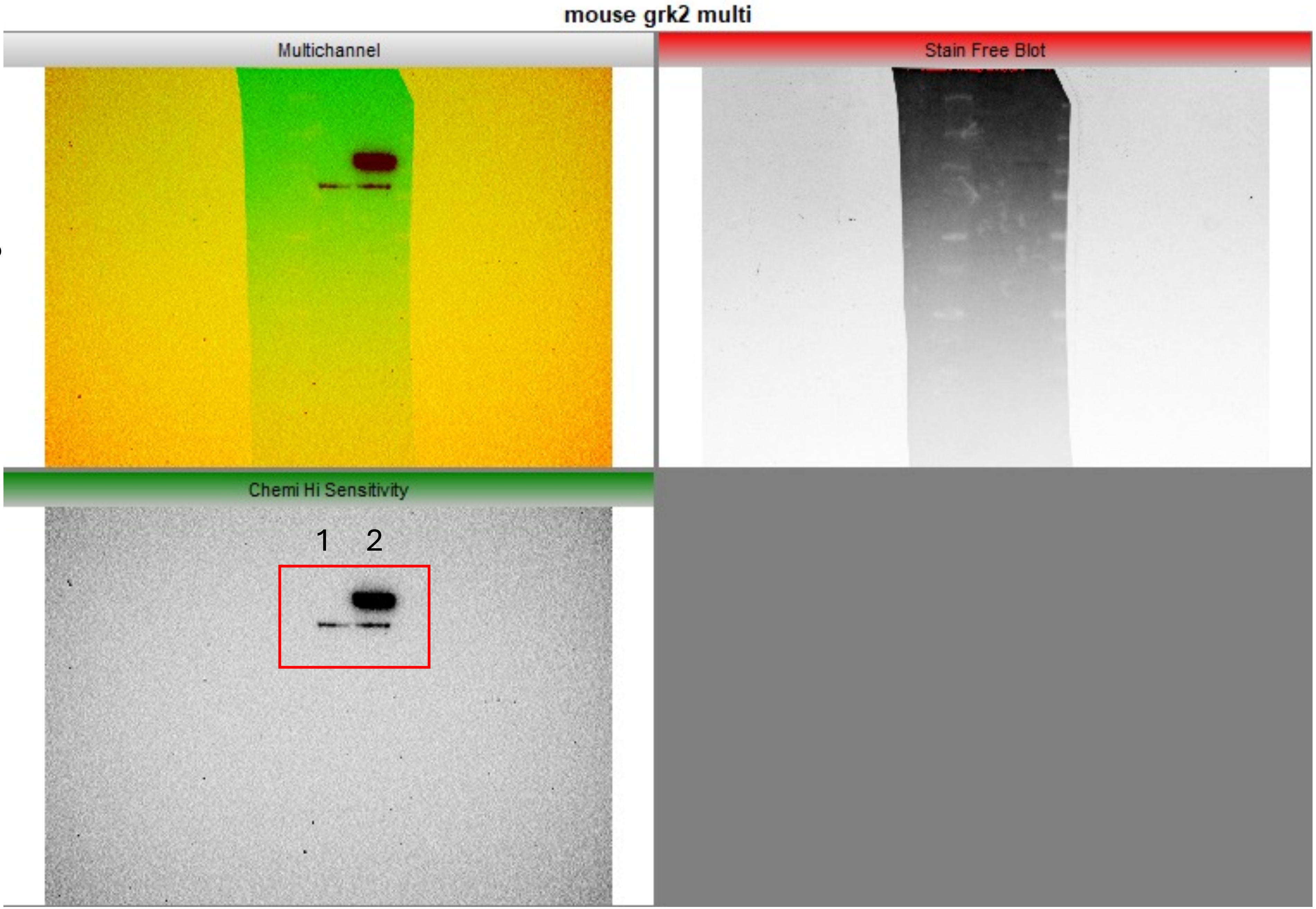

Fig S1 Total Protein

1 parental
2 GRK2-eGFP

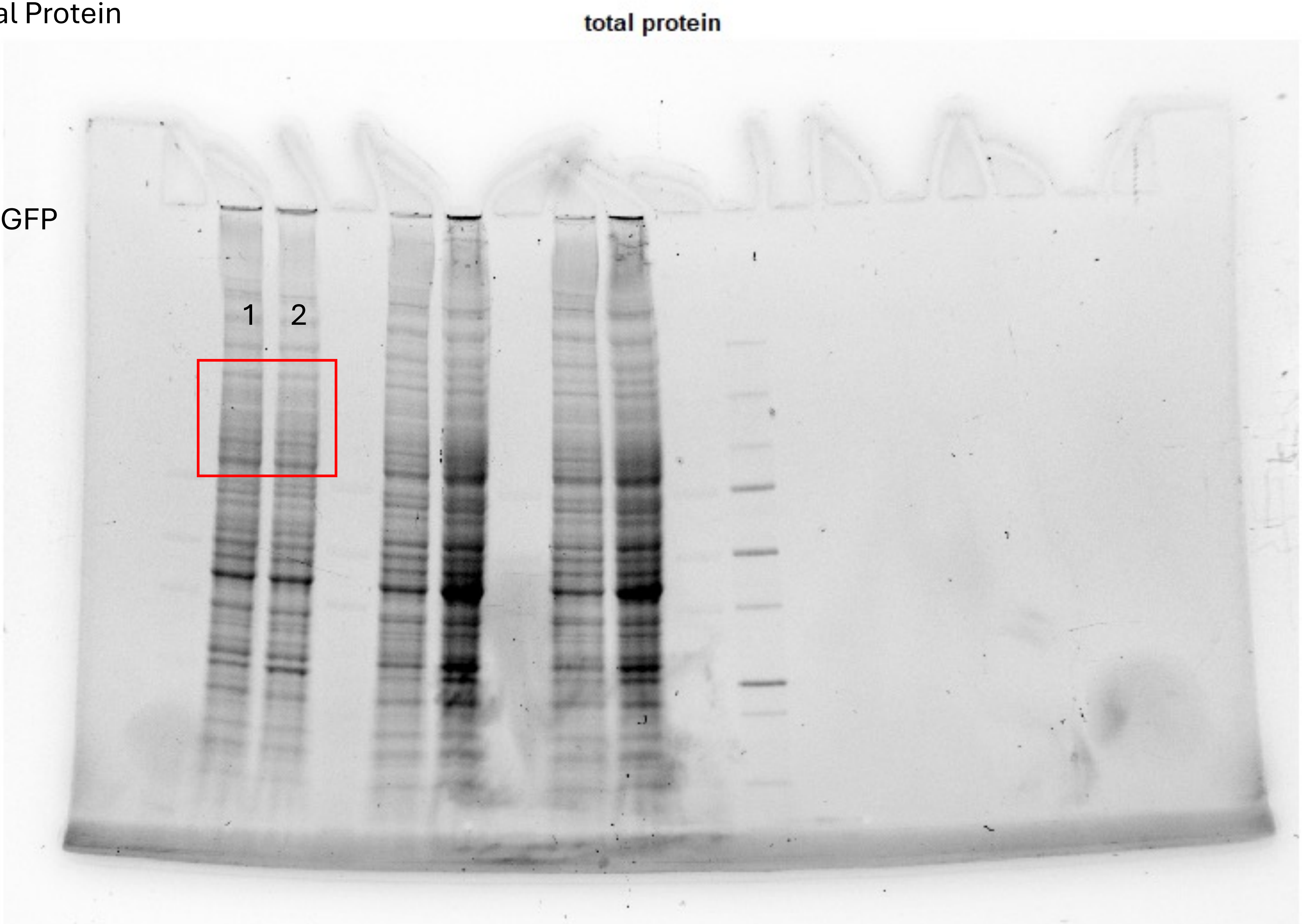

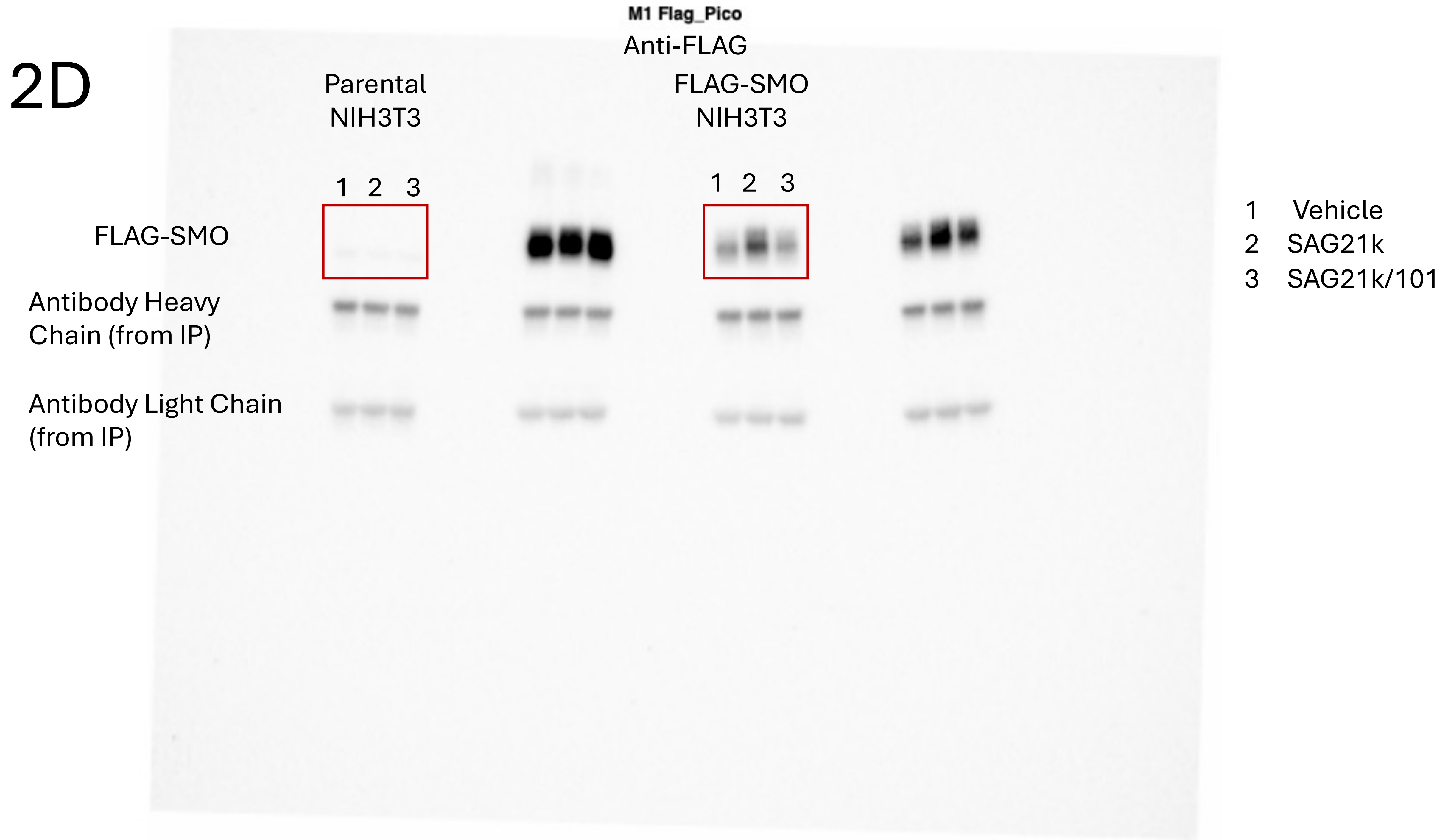
2D
M1 Flag_Pico
Anti-FLAG
Parental
NIH3T3
FLAG-SMO
NIH3T3
1 2 3
1 2 3
FLAG-SMO
Antibody Heavy
Chain (from IP)
Antibody Light Chain
(from IP)
1 Vehicle
2 SAG21k
3 SAG21k/101

2D

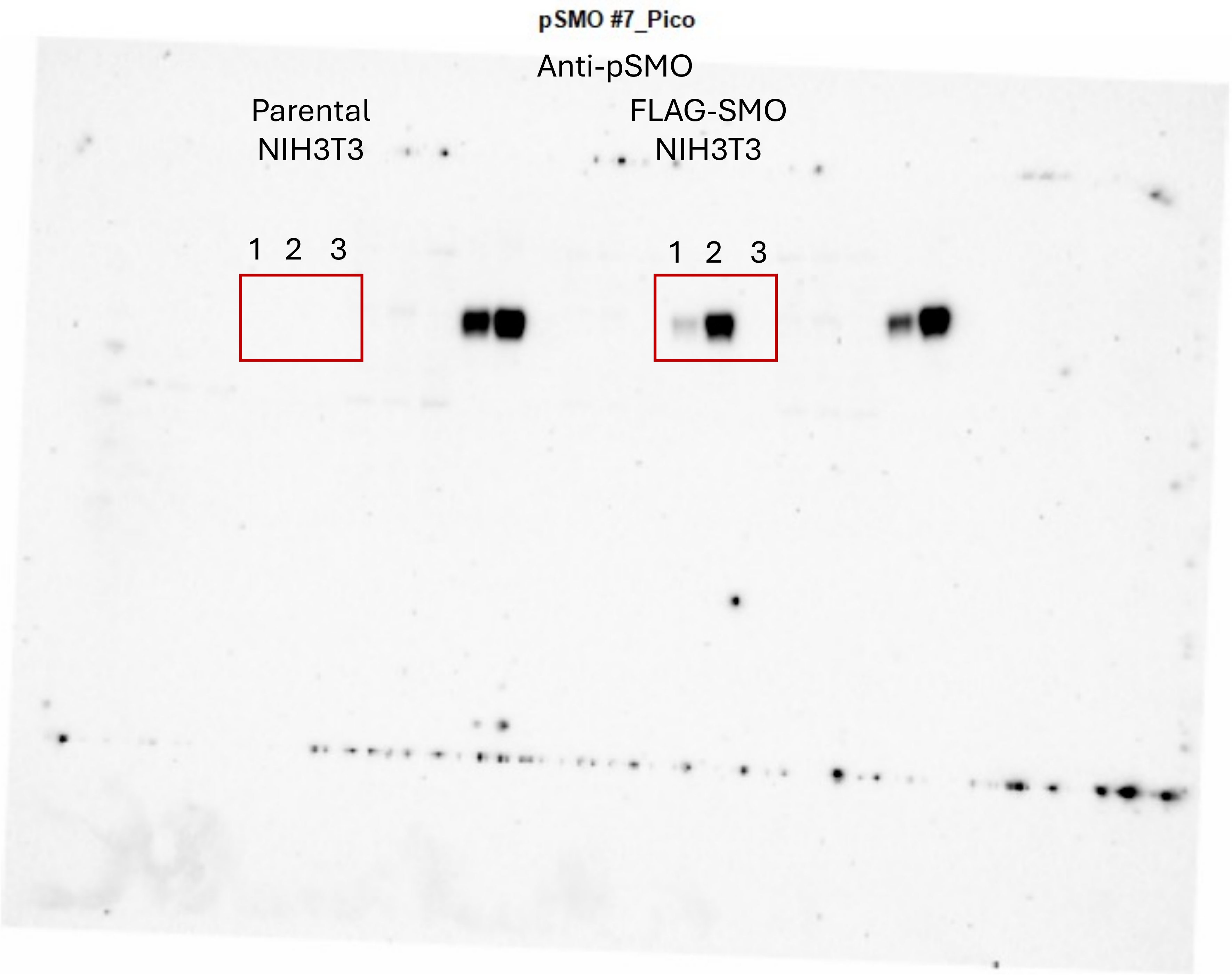

1 Vehicle
2 SAG21k
3 SAG21k/101

2D

Total protein

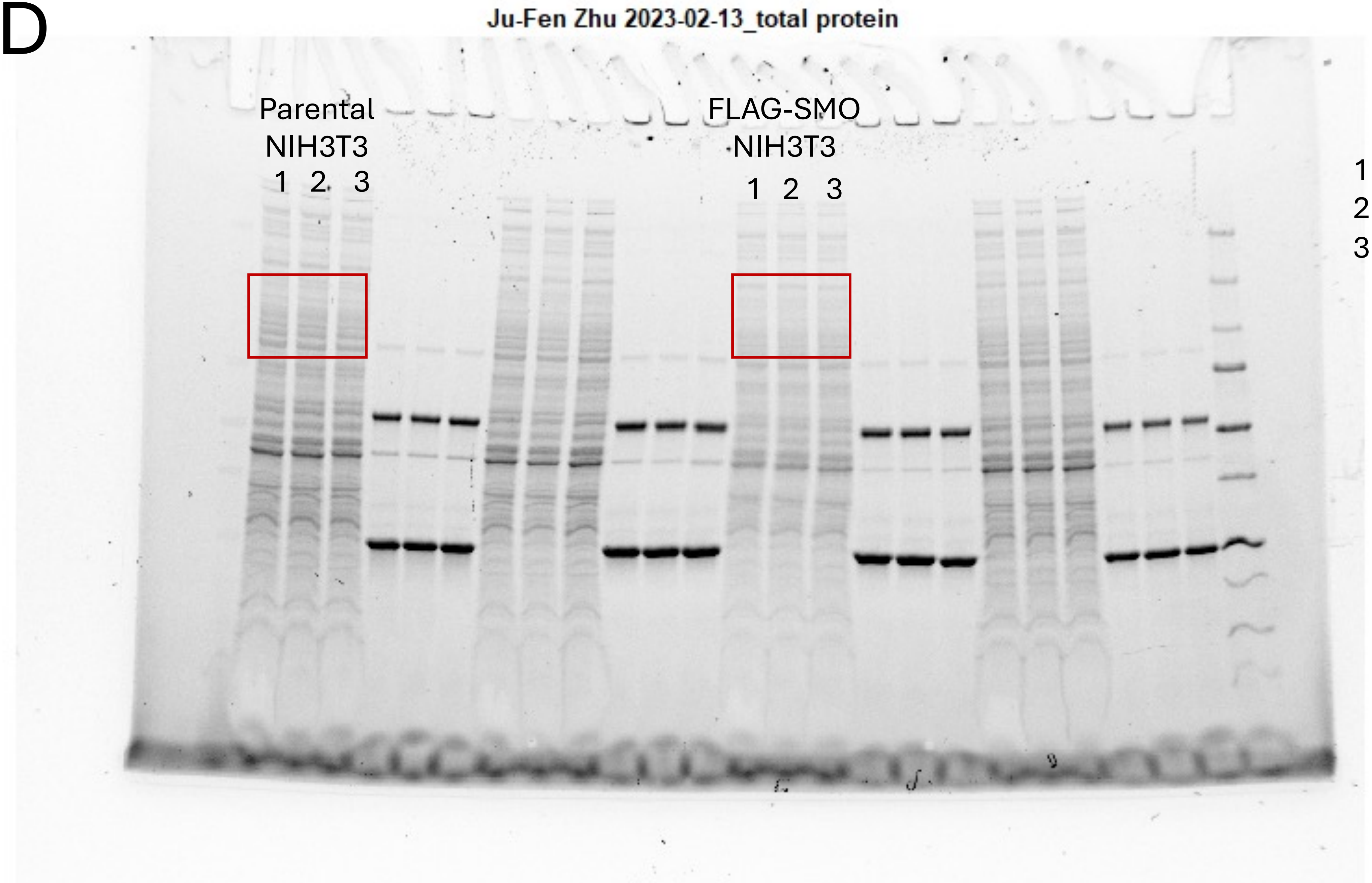

1 Vehicle
2 SAG21k
3 SAG21k/101

2E

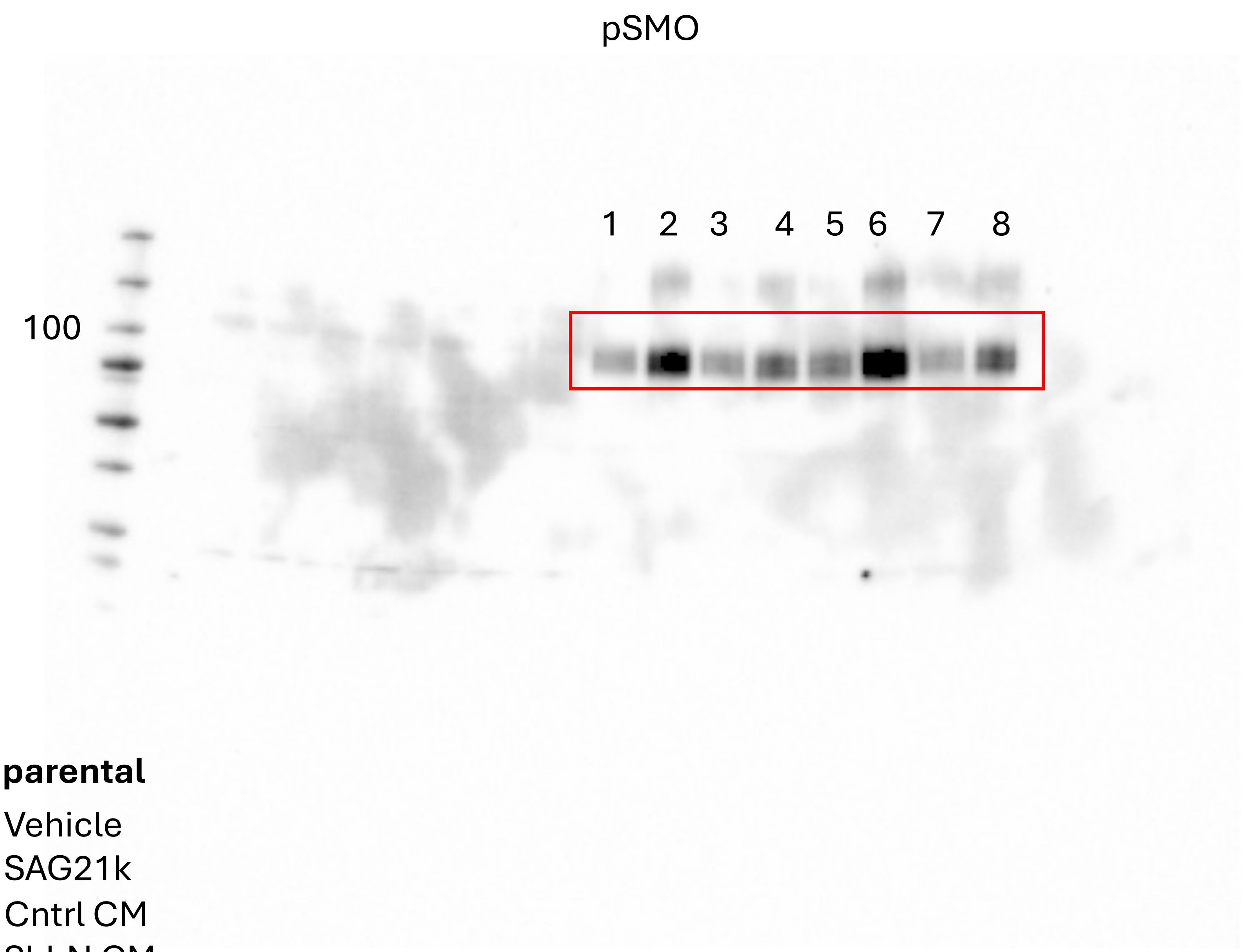

	Kif3a-/-		parental
1	Vehicle	5	Vehicle
2	SAG21k	6	SAG21k
3	Cntrl CM	7	Cntrl CM
4	ShhN CM	8	ShhN CM

2E

Kif3a-/-

1 Vehicle
2 SAG21k
3 Cntrl CM
4 ShhN CM

parental

5 Vehicle
6 SAG21k
7 Cntrl CM
8 ShhN CM

M1 FLAG

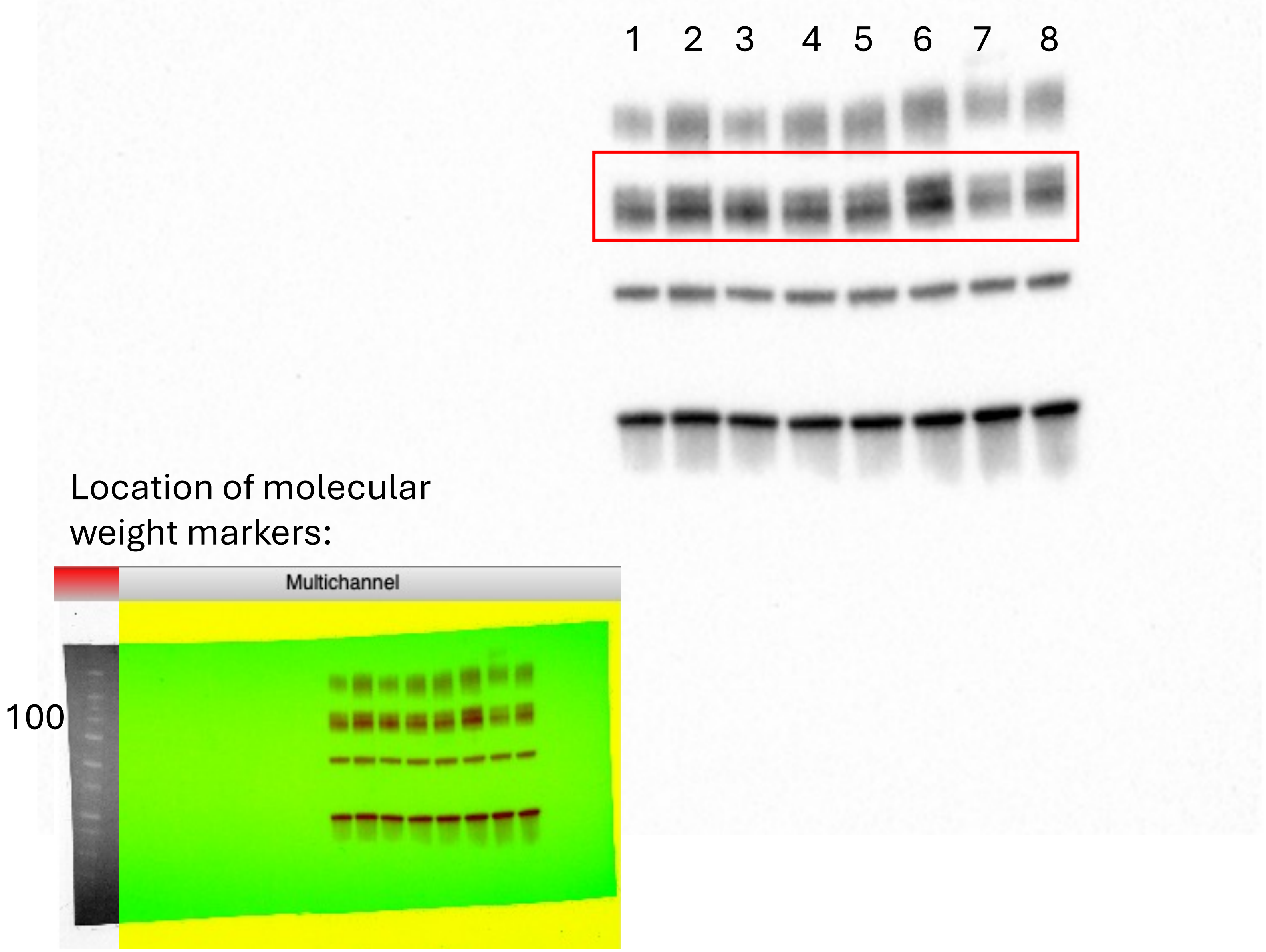

FLAG-SMO

Antibody Heavy Chain

Antibody Light Chain

2E

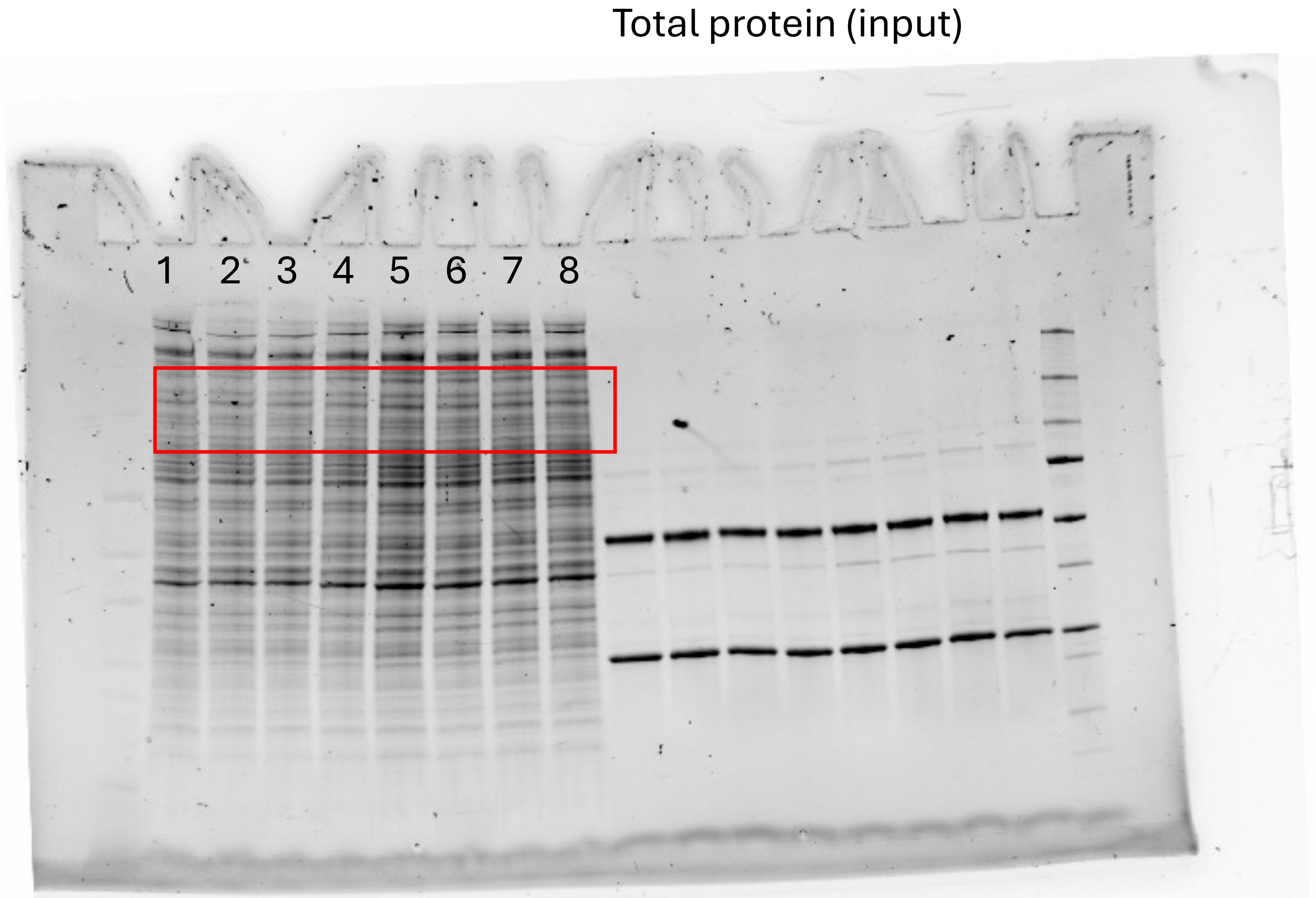

	Kif3a-/-		parental
1	Vehicle	5	Vehicle
2	SAG21k	6	SAG21k
3	Cntrl CM	7	Cntrl CM
4	ShhN CM	8	ShhN CM

S4A

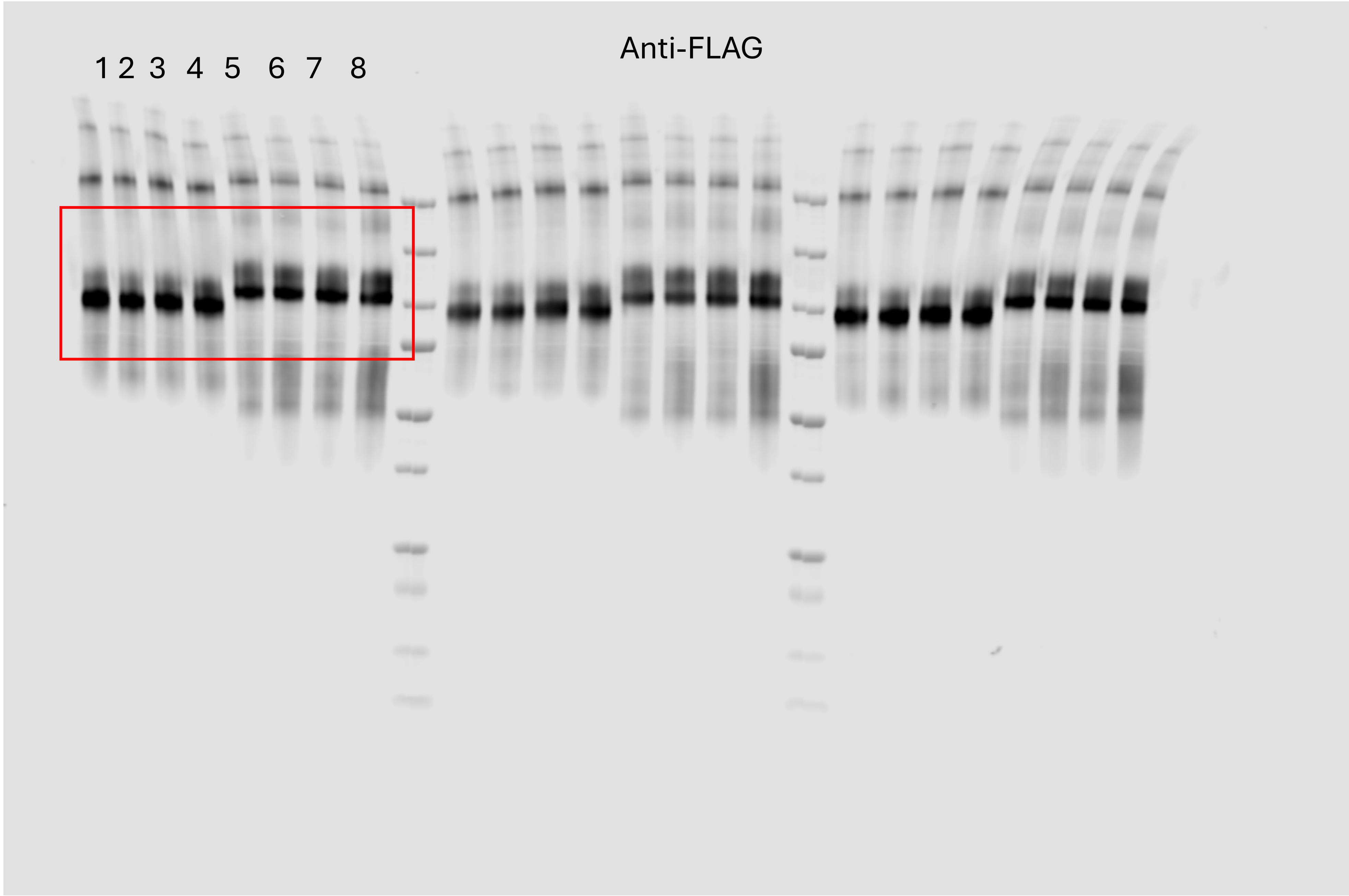

SMO-deltaCT

1 vehicle
2 KAADcyc
3 SAG21k
4 SAG21k/101

SMO

5 vehicle
6 KAADcyc
7 SAG21k
8 SAG21k/101

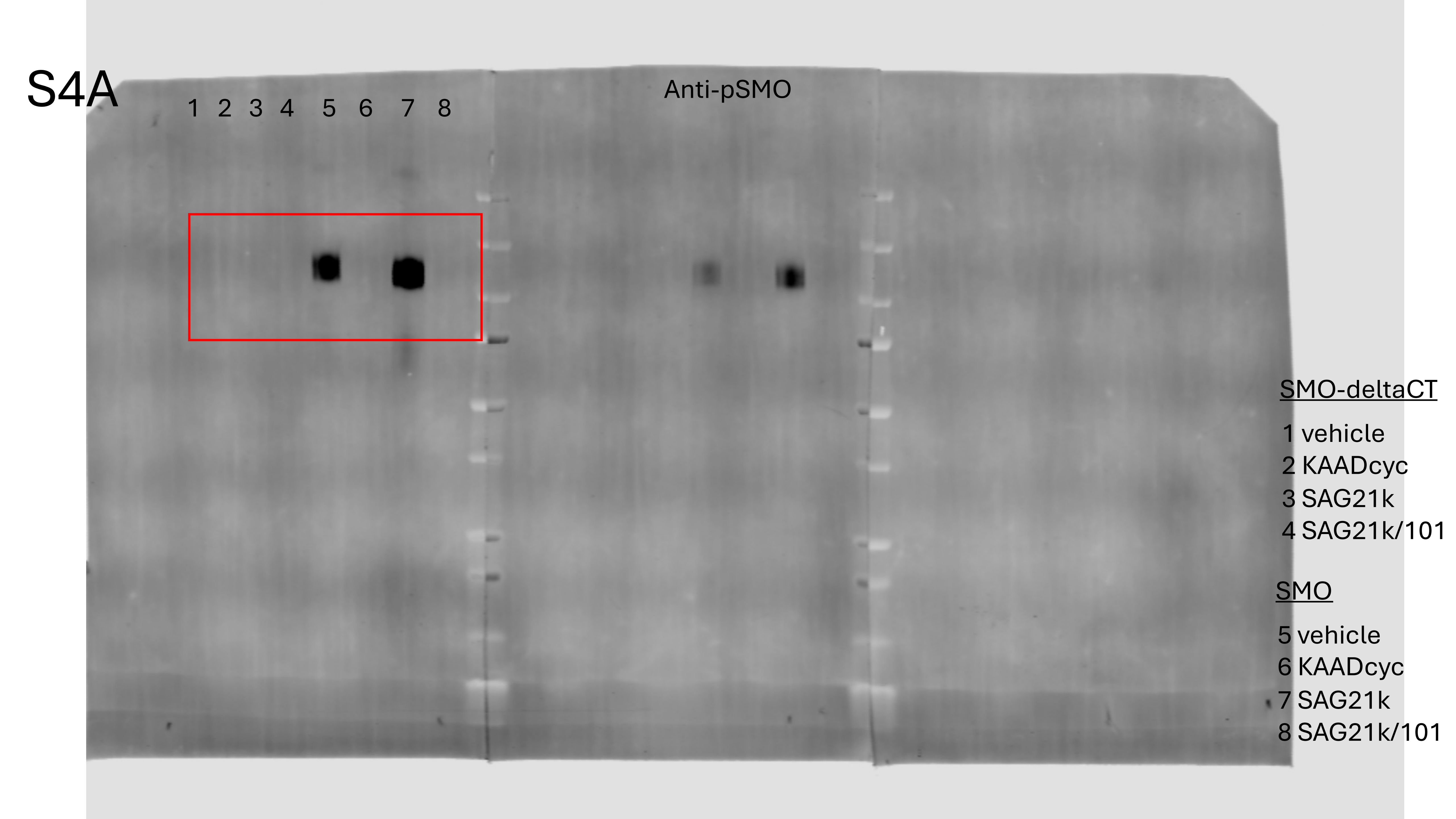
S4A
1 2 3 4 5 6 7 8
Anti-pSMO
SMO-deltaCT
1 vehicle
2 KAADcyc
3 SAG21k
4 SAG21k/101
SMO
5 vehicle
6 KAADcyc
7 SAG21k
8 SAG21k/101

S4B

1 No SMO
2 SMO, Vehicle
3 SMO, KAADcyc
4 SMO, cyclopamine
5 SMO, vismodegib
6 SMO, SANT-1
7 SMO, MBCD
8 SMO, SAG21k
9 SMO, purmorphamine
10 SMO, SAG21k/101
11 SMO, SAG21k/14as

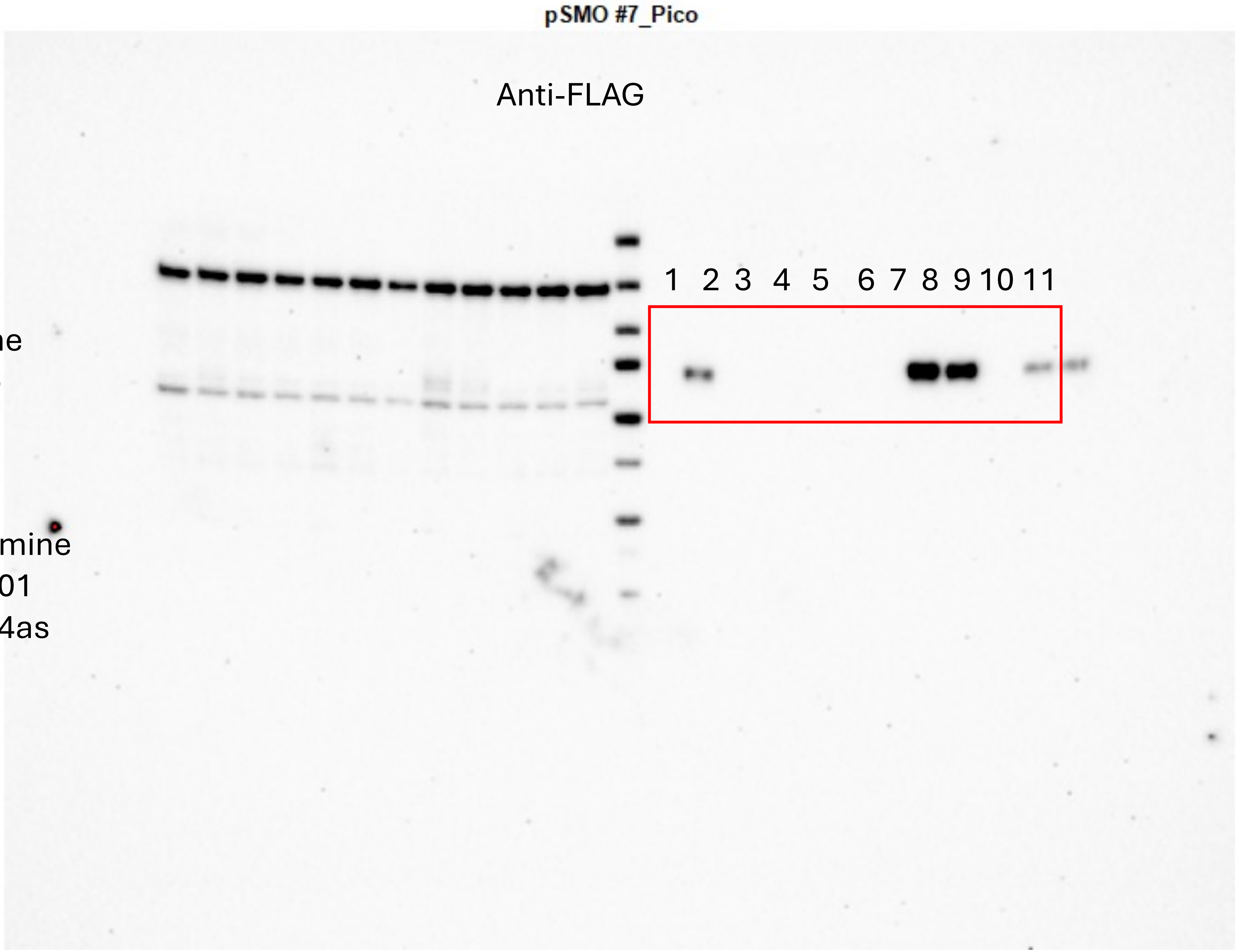

S4B

1 No SMO
2 SMO, Vehicle
3 SMO, KAADcyc
4 SMO, cyclopamine
5 SMO, vismodegib
6 SMO, SANT-1
7 SMO, MBCD
8 SMO, SAG21k
9 SMO, purmorphamine
10 SMO, SAG21k/101
11 SMO, SAG21k/14as

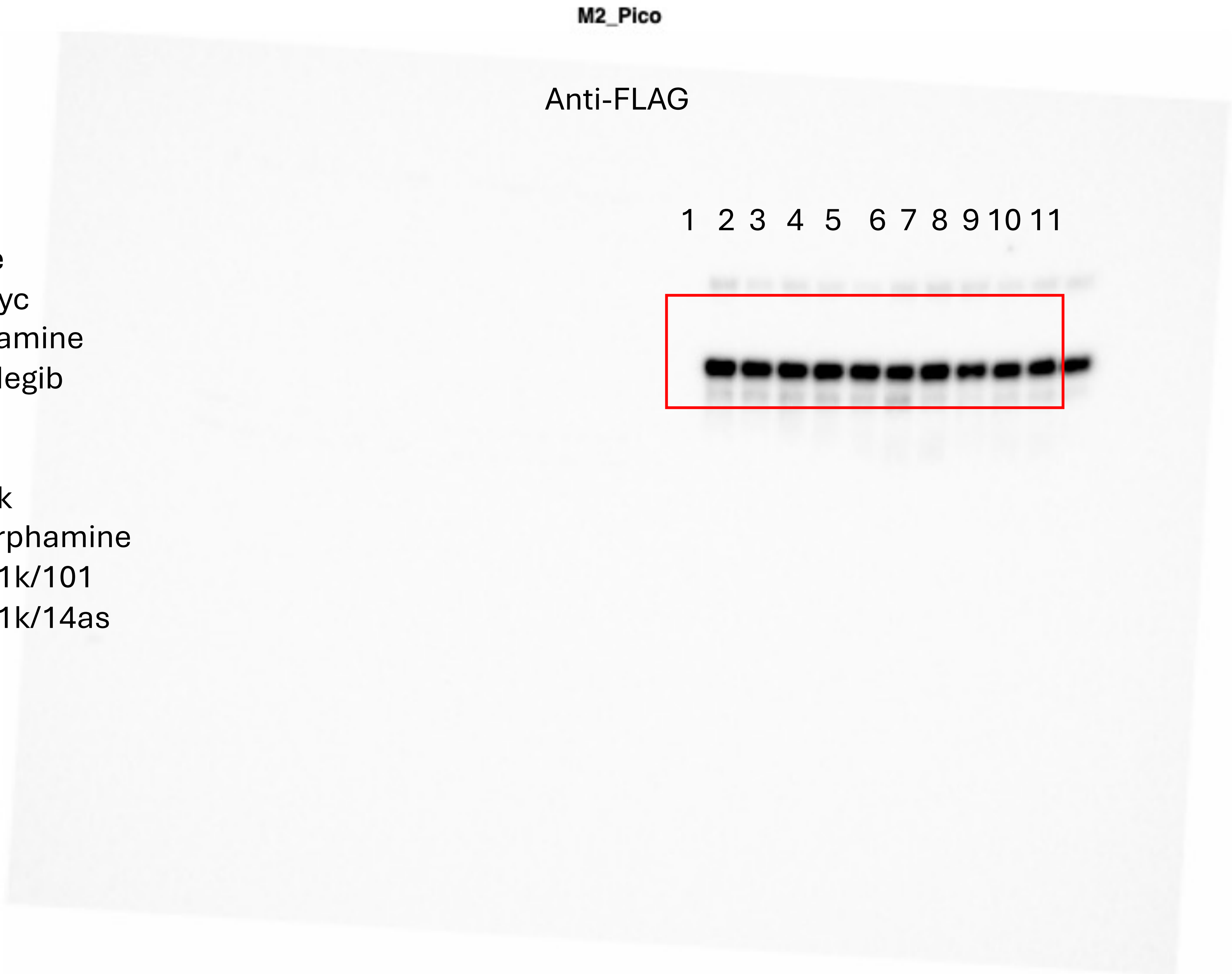

S5C

1 parental
2 clone #1
3 clone #2

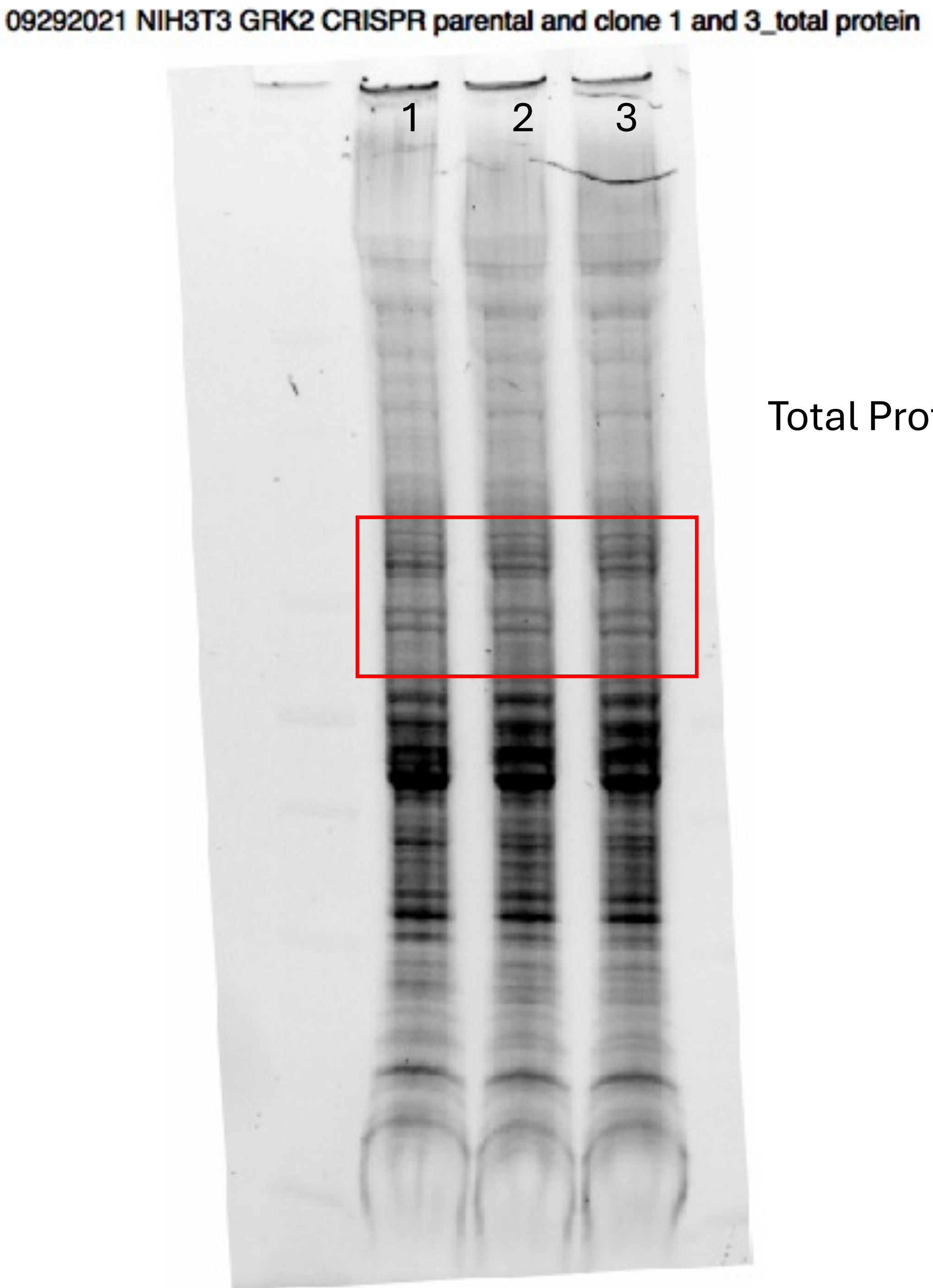

Total Protein Gel

S5C

1 parental
2 clone #1
3 clone #2

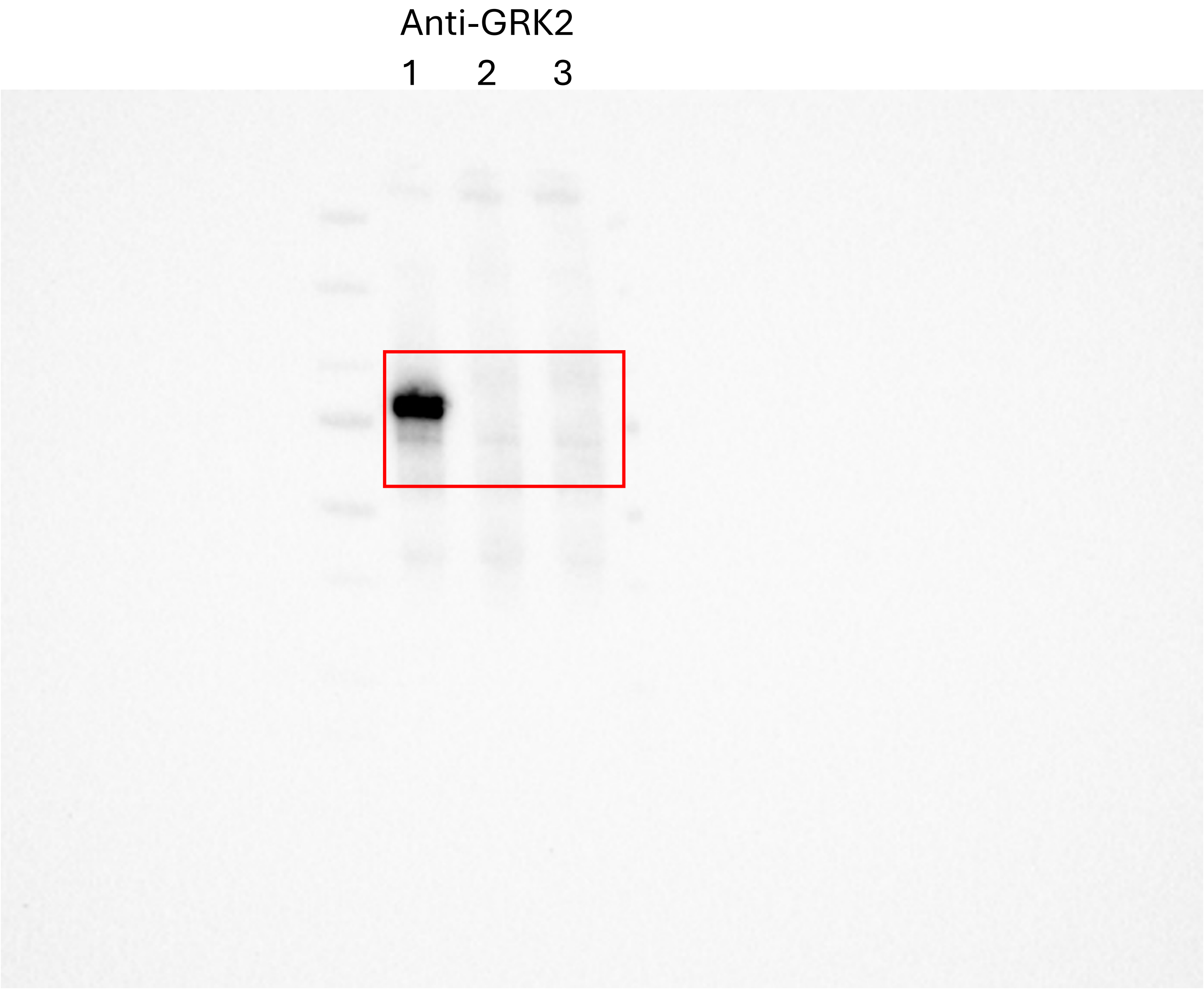

4B

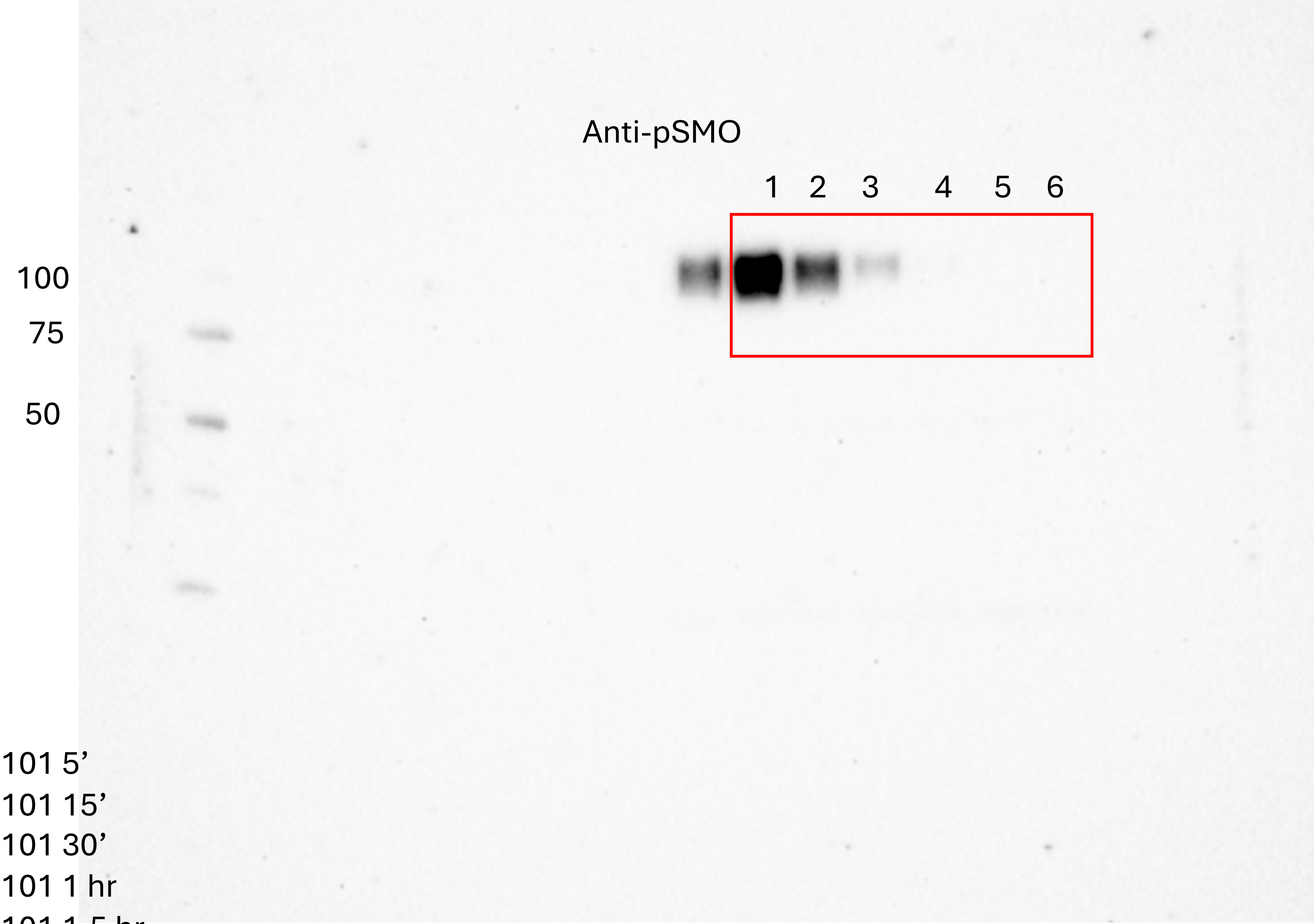

1 SAG21k
2 SAG21k + 101 5’
3 SAG21k + 101 15’
4 SAG21k + 101 30’
5 SAG21k + 101 1 hr
6 SAG21k + 101 1.5 hr

4B

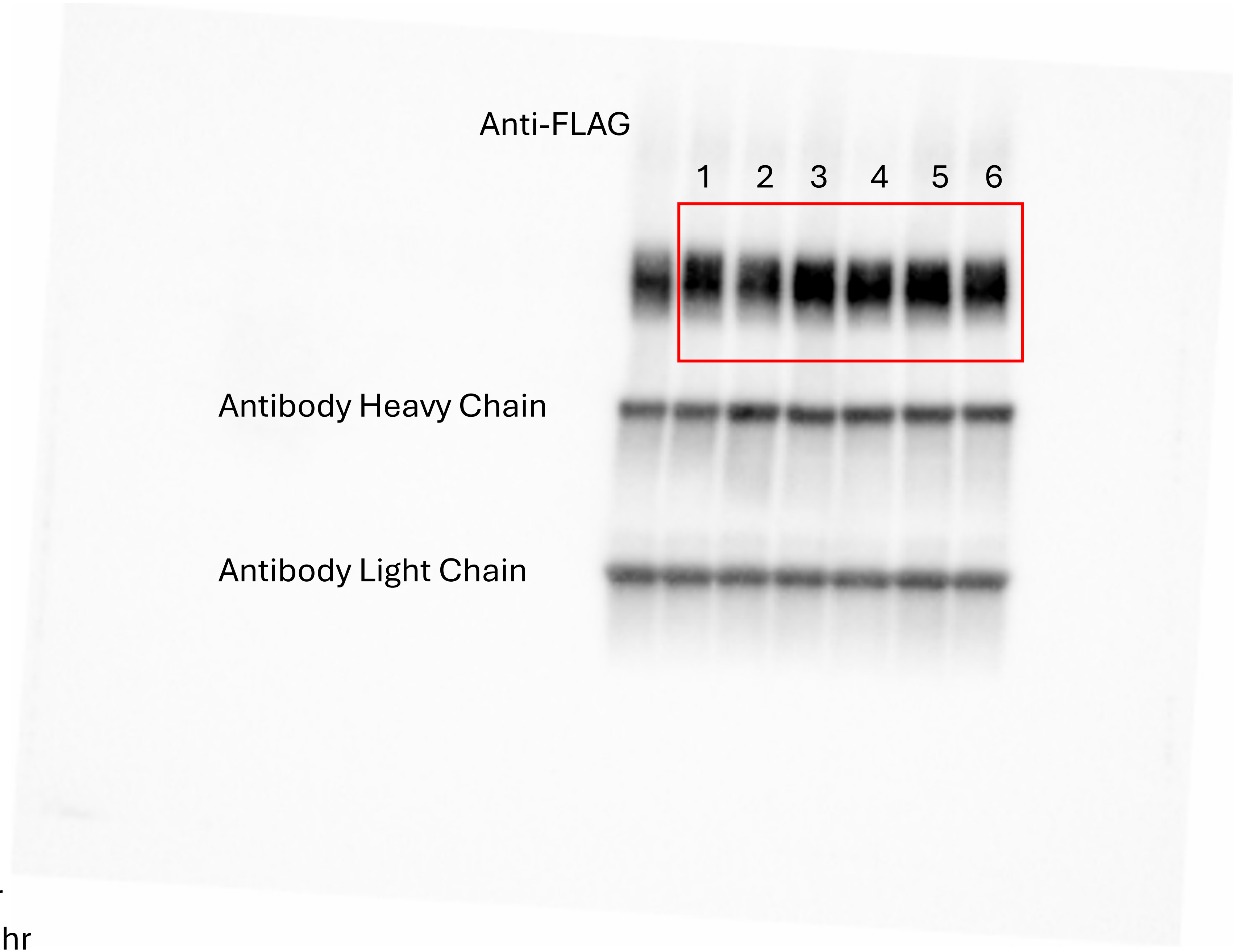

1 SAG21k
2 SAG21k + 101 5’
3 SAG21k + 101 15’
4 SAG21k + 101 30’
5 SAG21k + 101 1 hr
6 SAG21k + 101 1.5 hr

6B

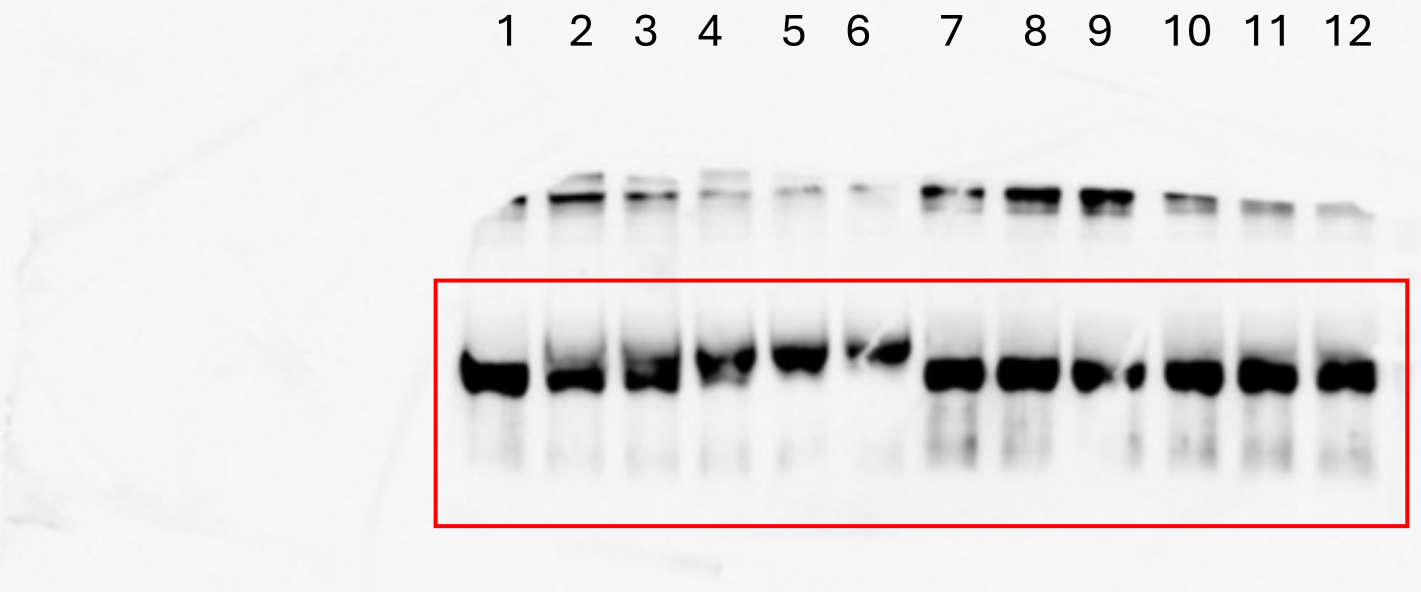

1 SAG21k, 0 min
2 SAG21k, 5 min
3 SAG21k, 10 min
4 SAG21k, 20 min
5 SAG21k, 40 min
6 SAG21k, 60 min
7 KAADcyc, 0 min
8 KAADcyc, 5 min
9 KAADcyc, 10 min
10 KAADcyc, 20 min
11 KAADcyc, 40 min
12 KAADcyc, 60 min

6C

input
1 PKA-C
2 PKA-C + SMO
3 PKA-C + pSMO
4 PKA-C + SMOΔCT

elution
5 PKA-C
6 PKA-C + SMO
7 PKA-C + pSMO
8 PKA-C + SMOΔCT

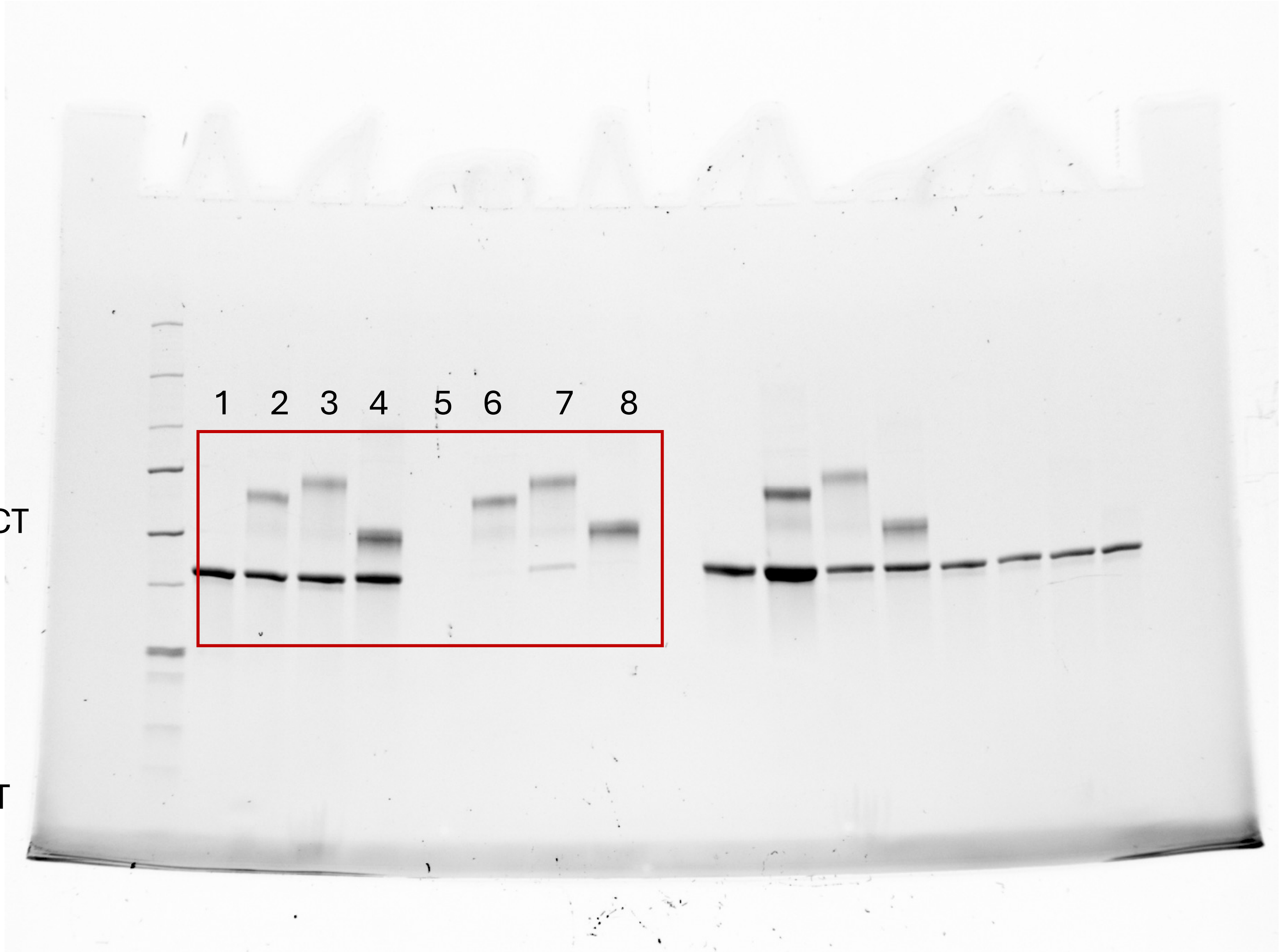

6D

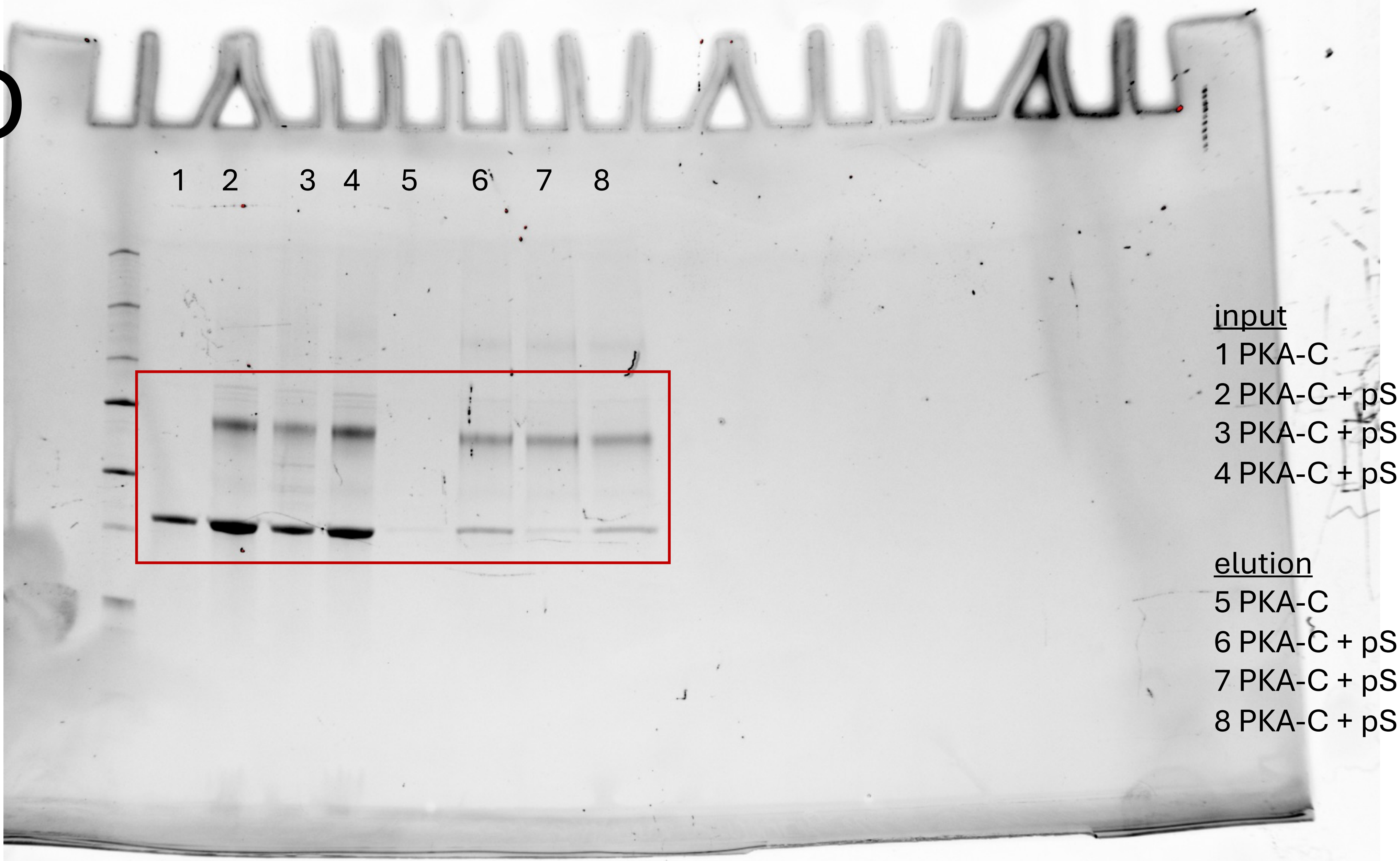

input
1 PKA-C
2 PKA-C + pSMO
3 PKA-C + pSMO + PKIa
4 PKA-C + pSMO + ctrl

elution
5 PKA-C
6 PKA-C + pSMO
7 PKA-C + pSMO + PKIa
8 PKA-C + pSMO + ctrl

S8A

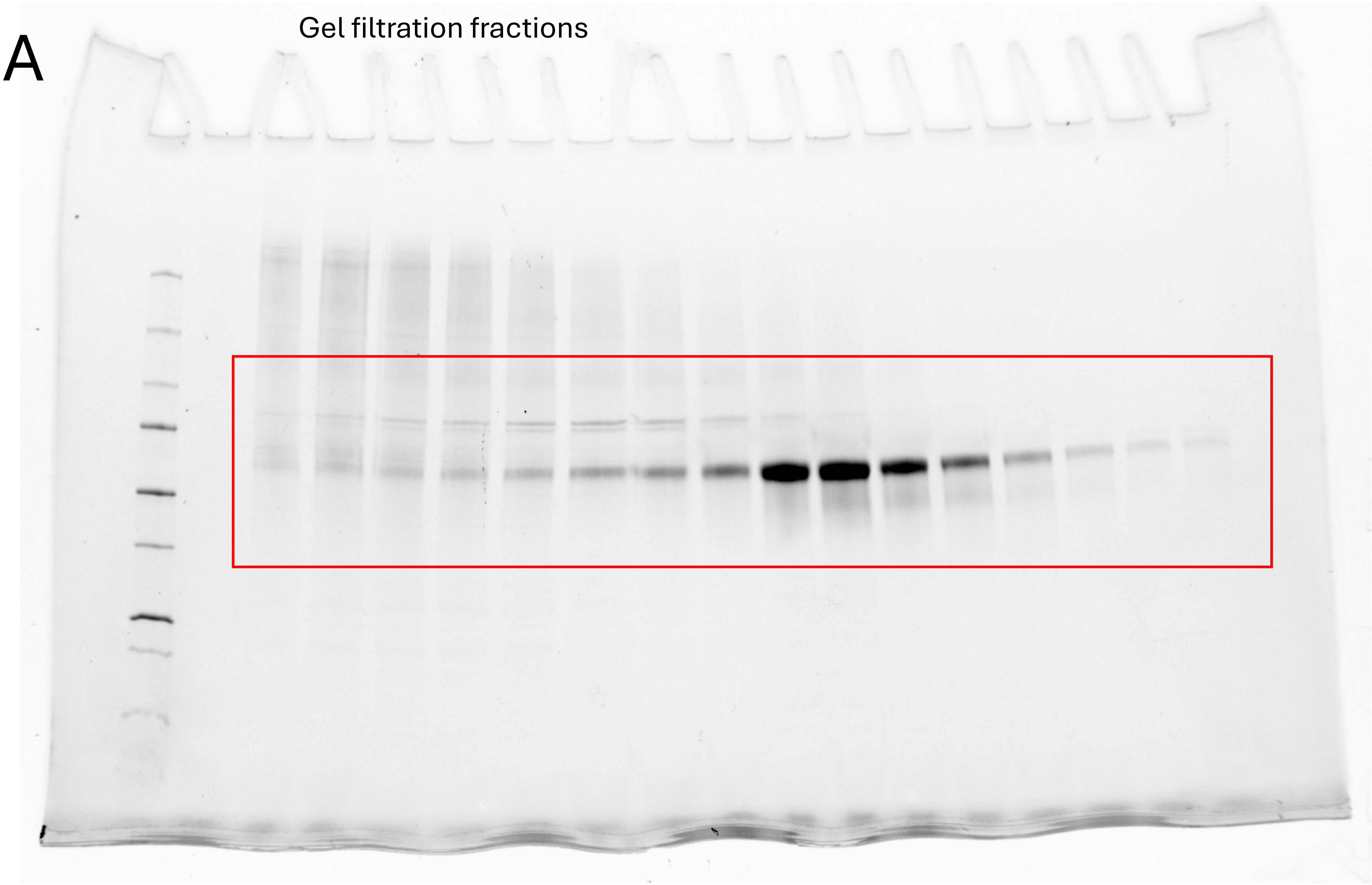

S8B

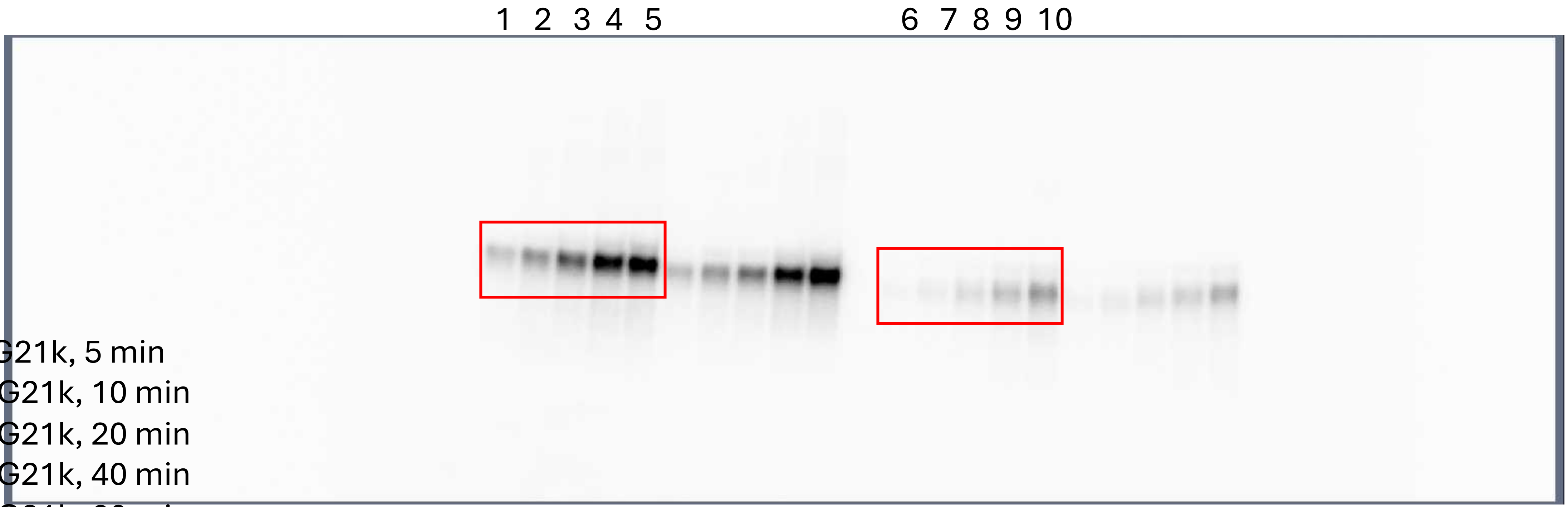

1 SAG21k, 5 min
2 SAG21k, 10 min
3 SAG21k, 20 min
4 SAG21k, 40 min
5 SAG21k, 60 min
6 KAADcyc, 5 min
7 KAADcyc, 10 min
8 KAADcyc, 20 min
9 KAADcyc, 40 min
10 KAADcyc, 60 min

SAG21k = SMO + agonist; KAADcyc = SMO + inverse agonist

S8C

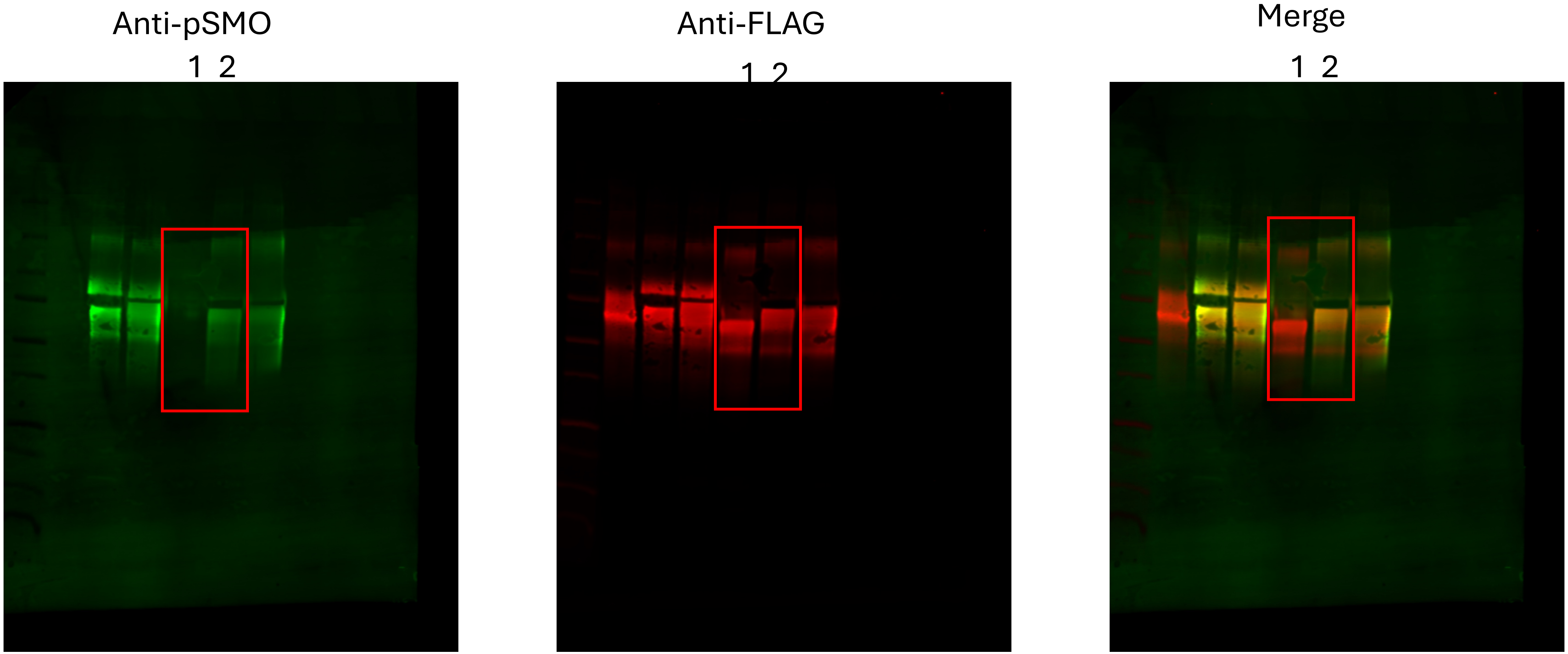

1 = SMO; 2 = SMO + GRK2

S8D

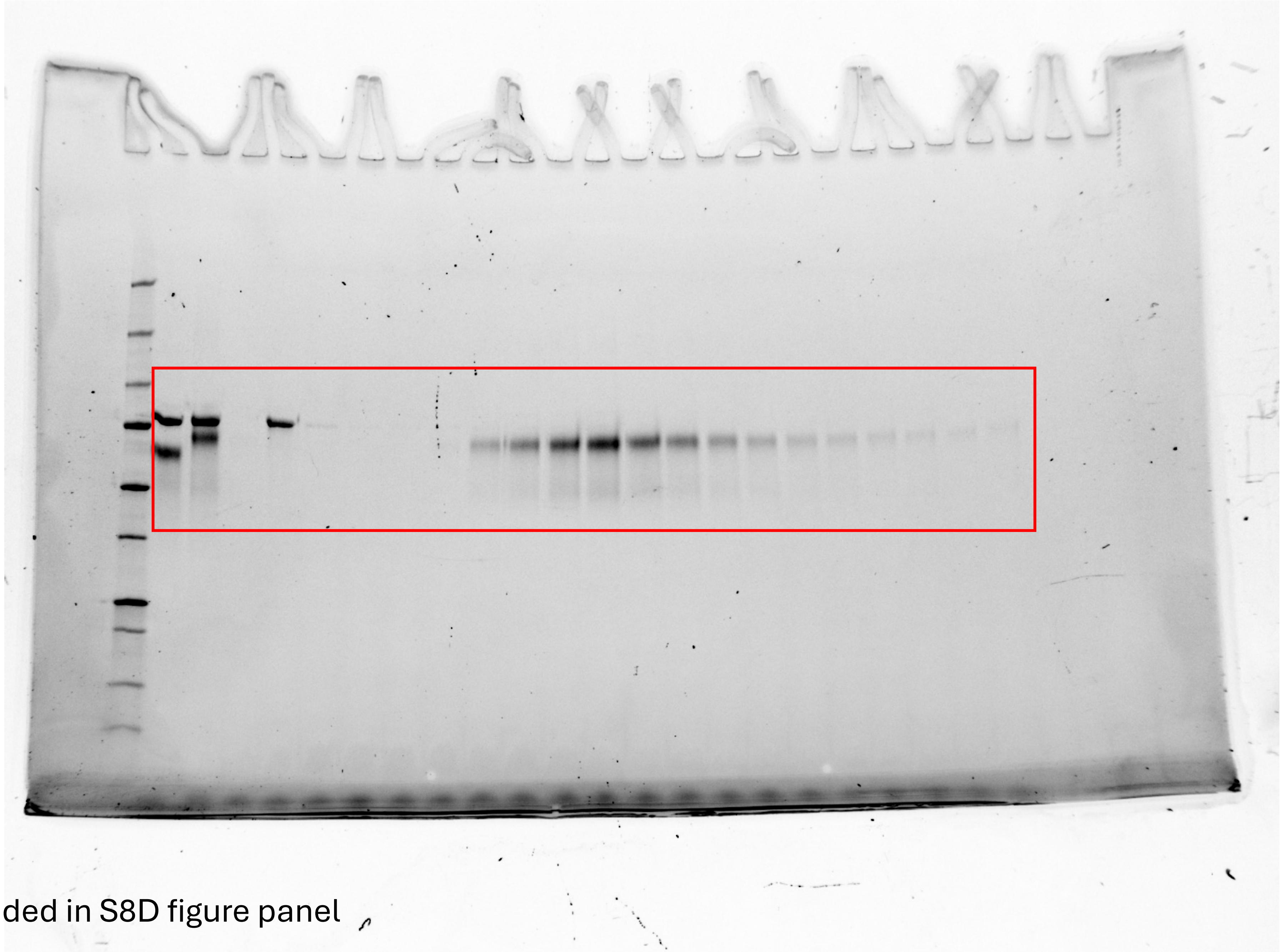

All labels provided in S8D figure panel
